# Electrochemical Boron Detection with Ferrocene and Catechol-Functionalized Cyclodextrin Inclusion Complex

**DOI:** 10.3390/ijms26094432

**Published:** 2025-05-07

**Authors:** Kai Sato, Hiroshi Kimoto, Takeshi Hashimoto

**Affiliations:** 1 Department of Materials and Life Sciences, Faculty of Science and Technology, Sophia University, 7-1 Kioi-cho, Chiyoda-ku, Tokyo 102-8554, Japan; k-sato-8u9@eagle.sophia.ac.jp; 2 Technical Development Division, Nomura Micro Science Co., Ltd., Atsugi 243-0021, Kanagawa, Japan; h-kimoto@nomura-nms.co.jp

**Keywords:** boron detection, current amplification, supramolecule, concerted oxidation, cyclodextrin, ferrocene, catechol, voltammetry

## Abstract

We demonstrate a rapid and sensitive boron detection method through current amplification mediated by supramolecular interaction. Oxidation peak currents obtained by cyclic voltammetry (CV) measurements of a ferrocene/catechol-functionalized *β*-cyclodextrin inclusion complex were amplified through an EC’ reaction (where EC’ denotes an electrochemical step followed by a catalytic chemical step). However, the amplified current was decreased by boric acid (the primary form of boron in water) addition at pH 8.6 owing to interactions of boron with the *cis*-diol structure of dihydroxybenzoic acid-*β*-cyclodextrin and ferrocene for ester formation. We determined the optimum CyD functionalization sites and measurement conditions and obtained a limit of detection of 0.16 mg B L^−1^ for ferrocene/3,4-dihydroxybenzoic acid-*β*-cyclodextrin (Fc/3,4-DHBA-*β*-CyD). The binding constant (assuming a 1:1 binding model) for the interaction between Fc/3,4-DHBA-*β*-CyD and boric acid was estimated to be approximately 1500 M^−1^. Boron concentrations in spiked real samples showed good recoveries and linear calibration curves. The electrochemical response of this system was not significantly affected by the presence of other anions or cations. We also found that an aqueous solution of 3,4-DHBA-*β*-CyD remained stable for at least 112 days.

## 1. Introduction

Boron is a trace element necessary for human health and plant growth [1,2]. However, the excessive intake of this metalloid may have deleterious effects [3,4,5]. In 2011, the World Health Organization established the maximum boron concentration in drinking water at 2.4 mg B L^−1^ [6,7]. This limit is further reduced to 1.0 mg B L^−1^ in Japan [8] and the European Union [9]. Because seawater contains approximately 4 mg B L^−1^ [10,11], it is crucial to monitor boron concentration in drinking water generated by desalination plants [11]. In addition, because excess boron can negatively impact plants [3], boron levels in irrigation water and soil must be assessed [12,13]. Boron is widely used in industry [14], pharmaceuticals [15], and nuclear power plants [12,16]. In Japan, the limit for boron and related compounds discharged into public waters, excluding marine areas, is set at 10 mg B L^−1^ [17,18]. Hence, boron concentrations in wastewater must be closely monitored. Semiconductor manufacturing requires ultrapure water whose boron concentrations are of the order of ng B L^−1^ to avoid introducing p-type dopants that can lead to circuit leakage [12]. Although ultrapure water is generally obtained by reverse osmosis (RO), it is difficult to remove boron from water altogether using this technology [19,20]. In medicine, the advent of boron neutron capture therapy for treating cancer has made monitoring blood boron concentrations a necessity [21,22]. These circumstances underscore the importance of boron analysis.

Boron detection methods can be classified into two categories: (1) an instrumental method (IM) that involves the direct measurement of boron atoms, and (2) a chemical method (CM) that utilizes the interaction between boron and a reagent molecule. The inductively coupled plasma (ICP) method [23], which includes ICP-AES and ICP-MS, is categorized in the former and can be used for sensitive boron detection [24,25] in seawater desalination plants and semiconductor manufacturing. However, it requires expensive specialized equipment and complex analytical procedures [26]. In addition, the strong boron memory effect requires thoroughly cleaning the chambers of ICP instruments [26].

On the other hand, many CMs detect the spectroscopic or electrochemical response of a reagent molecule through the ester formation between boric acid and a diol moiety of the reagent molecule [27,28]. For example, yellow curcumin forms a 2:1 complex with boric acid, called rosocyanin [29,30,31], and this method for determining boron is known as the curcumin method. However, curcumin is prone to degradation by light [32]. Azomethine H is a colorimetric reagent that forms a red complex with boron [33,34]. However, this reaction has several problems, including the coloration of the blank solution with time and a long reaction time. K. Takehara et al. applied azomethine H to voltammetric measurements [35]. M. Kajiwara et al. proposed a highly sensitive and rapid voltammetric determination of boron using 5-fluorosalicylaldehyde (F-SA) [36]. T. Fujimori et al. developed an electrochemical method for boron determination using tiron [37]. E. Nakano et al. developed a boron detection method using a ruthenium(II) complex, [Ru^II^(bpy)_2_(dhphen)]^2+^ (bpy = 2,2′-bipyridine, dhphen = 5,6-dihydroxy-1,10-phenanthroline), as a fluorescent reagent for boron detection [38]. We have reported an electrochemical boron detection method using [Ru^III^(acac)_2_(H_2_thap)] (acac = acetylacetonato ion, H_2_thap = 2′,3′,4′-trihydroxyacetophenonate (gallacetophenonate) ion) [39]. Other boron detection methods utilizing carminic acid [40,41], alizarin red S [42], beryllon III [43,44], and gallacetophenone [28] have been reported as well. Most CMs employ the reaction between boron and a diol moiety, except the methylene blue extractive absorption spectrophotometric method. In this method, hydrofluoric acid and sulfuric acid are added to the sample to convert boric acid into tetrafluoroboric acid, which forms an aggregate with methylene blue. This aggregate is extracted into 1,2-dichloroethane, and the absorbance is measured for boron determination [45,46,47]. This method has several drawbacks; it requires numerous complicated steps, uses hazardous reagents, and is susceptible to interference by chromate ions and turbid components. As described above, most CMs are based on a simple 1:1 binding between boron and a reagent molecule.

In our previous study [48], we reported current amplification in the voltammetry of catechol-functionalized *β*-CyD in the presence of ferrocene (Fc). The amplified current originated from an EC’ reaction that features a concerted oxidation through steric interactions involving the inclusion of Fc into the functionalized CyD. Experiments showed that the amplified current was decreased in the presence of boric acid, because boric acid bound to the catechol moiety, inhibiting the concerted oxidation. In this study, we applied this phenomenon to detect boron in aqueous solutions.

## 2. Results

All the experiments in this study were performed on aqueous solutions of supramolecular complexes dissolved in a 10% CH_3_OH/90% H_2_O (*v*/*v*). The results of each experiment are shown below.

### 2.1. Effect of CyD Functionalization Site

First, we investigated the structural effects of the CyD functionalization site on boric acid detection. Various *β*-CyDs with different functionalization sites (Figure 1; a total of ten types including one type without an OH group, three types with one OH group, five types with two OH groups, and one type with three OH groups) were synthesized (Figures S1–S21, Schemes S1–S6, Table S1). And then, the electrochemical response of the inclusion complexes of Fc with these *β*-CyDs and 3,4-DHBA-*β*-CyD alone to boron was investigated to determine the structure that shows the largest response (Figure 2 and Figure 3). The pH was set to 8.6 because current amplification was observed at pH 8.6 in our previous report [48]. The inclusion of Fc by each functionalized CyD was confirmed by ^1^H NMR studies (Figure S22).

**Figure 2:**
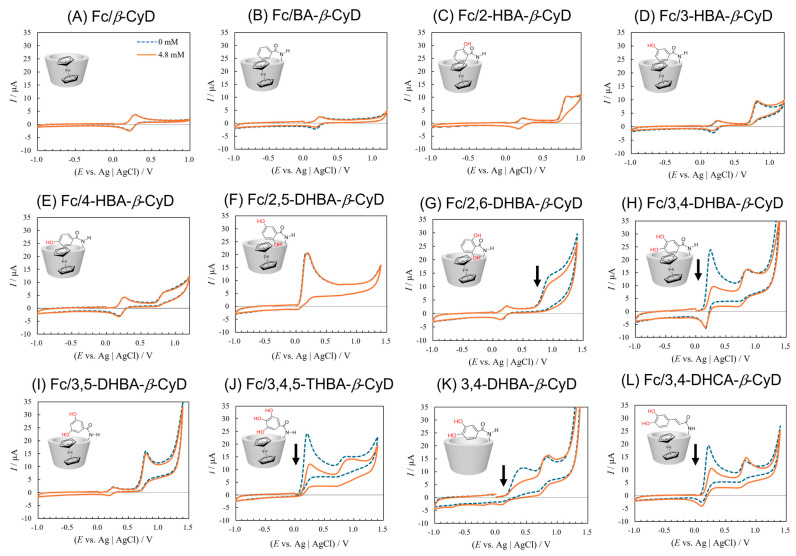
CV data acquired from solutions containing the functionalized CyD complexes (**A**–**L**) in 10% CH_3_OH/90% H_2_O (*v*/*v*) with 4.8 mM boron (red solid lines) or without boron (blue broken lines) at pH 8.6 and r.t. [Fc] = 0.5 mM, [functionalized *β*-CyD] = 1.5 mM, [NaCl] = 0.1 M, [phosphate] = 50 mM, and scan rate = 0.1 V s^−1^. WE: sGCDE (*ϕ* = 3.0 mm), RE: Ag|AgCl _3M NaCl aq._, and CE: Pt coil.

**Figure 3:**
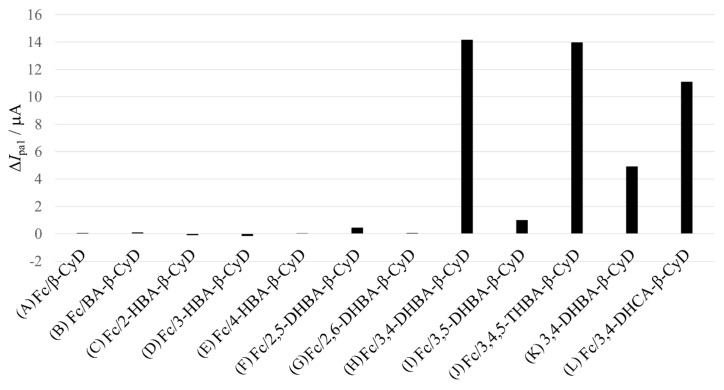
Responses of functionalized CyD complexes to boron calculated from Figure 2. The value of Δ*I*_pa1_ was calculated as (*I*_pa1 without boric acid_) − (*I*_pa1 with 4.8 mM of boric acid_).

Cyclic voltammetry (CV) data were obtained for solutions containing a boron concentration of 0 or 4.8 mM. In CV measurements without boron, current amplification was observed for Fc/2,5-DHBA-*β*-CyD (Figure 2F), Fc/3,4-DHBA-*β*-CyD (Figure 2H), Fc/3,4,5-THBA-*β*-CyD (Figure 2J), and Fc/3,4-DHCA-*β*-CyD (Figure 2L), having two or more OH groups that could be oxidized into quinone. The amplified currents of inclusion complexes except Fc/2,5-DHBA-*β*-CyD were markedly decreased in the presence of boron. The largest response was observed for Fc/3,4-DHBA-*β*-CyD (Figure 2H and Figure 3). Thus, Fc/3,4-DHBA-*β*-CyD was investigated further.

### 2.2. Determination of Optimum pH

**Figure 4:**
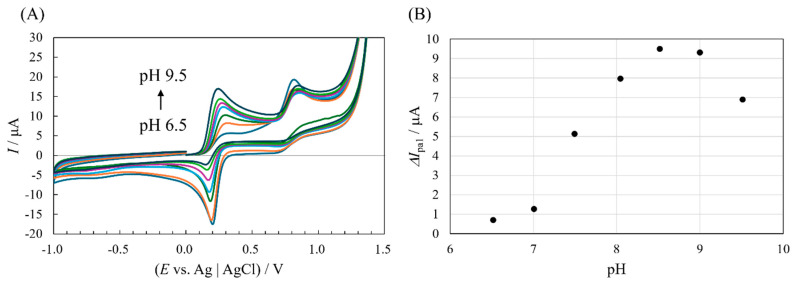
(**A**) CVs in various pH solutions containing Fc/3,4-DHBA-*β*-CyD with boron (*c*_B_ = 5 mM) in 10% CH_3_OH/90% H_2_O (*v*/*v*) at r.t. [Fc] = 0.5 mM, [3,4-DHBA-*β*-CyD] = 1.5 mM, [NaCl] = 0.1 M, [phosphate] = 50 mM, and scan rate = 0.1 V s^−1^. WE: sGCDE (*ϕ* = 3.0 mm), RE: Ag|AgCl _3M NaCl aq._, and CE: Pt coil. (**B**) Plot of Δ*I*_pa1_ (= (*I*_pa1 without boric acid_) − (*I*_pa1 with 5 mM of boric acid_)) as a function of pH.

In the presence of 5 mM of boron, CV data were obtained for Fc/3,4-DHBA-*β*-CyD in 10% CH_3_OH/90% H_2_O (*v*/*v*) at various pH values (Figure 4A). Only one oxidation peak was observed at 0.79 V at pH 6.5, whereas a new peak appeared at 0.28 V at pH 7.0. The current value for this first oxidation peak (*I*_pa1_) increased and the peak potential was negatively shifted with increasing pH. As previously reported [48], similar CV changes were observed in the absence of boron. However, the increase in Ipa1 with increasing pH in the presence of boron was less than that in the absence of boron (Figure S23). The *I*_pa1_ values obtained with and without boron are plotted against pH in Figure S23. The trend exhibited by *I*_pa1_ with increasing pH differed with and without boron, and the greatest variation was noted in the vicinity of pH 8.6 (Figure 4B).

### 2.3. Response of Fc/3,4-DHBA-β-CyD to Boron

#### CVs at Various Boron Concentrations

**Figure 5:**
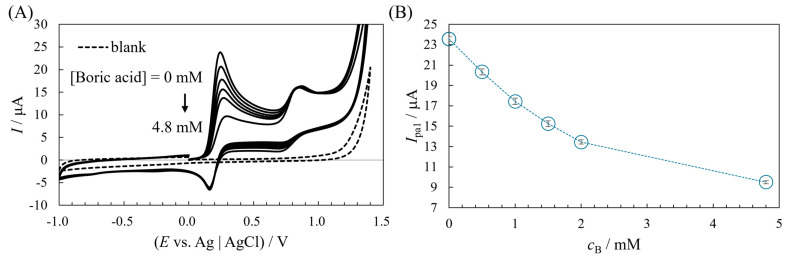
(**A**) CV of solutions containing Fc/3,4-DHBA-*β*-CyD in 10% CH_3_OH/90% H_2_O (*v*/*v*) with boron concentrations ranging from 0 to 4.8 mM at pH 8.6 and r.t. [Fc] = 0.5 mM, [3,4-DHBA-*β*-CyD] = 1.5 mM, [NaCl] = 0.1 M, [phosphate] = 50 mM, and scan rate = 0.1 V s^−1^. WE: sGCDE (*ϕ* = 3.0 mm), RE: Ag|AgCl _3M NaCl aq._, and CE: Pt coil. The broken line is the result of a blank measurement. (**B**) Plot of *I*_pa1_ as a function of *c*_B_ (*n* = 3).

CVs of Fc/3,4-DHBA-*β*-CyD in 10% CH_3_OH/90% H_2_O (*v*/*v*) at pH 8.6 were performed at boron concentrations ranging from 0 to 4.8 mM to evaluate the electrochemical responses. No peaks were generated in a solution containing a 50 mM phosphate buffer and 0.1 M NaCl in the absence of Fc/3,4-DHBA-*β*-CyD (Figure 5A). In the presence of Fc/3,4-DHBA-*β*-CyD, oxidation peaks were observed at 0.25 V (*I*_pa1_) and 0.85 V (*I*_pa2_). According to our previous report [48], the first oxidation peak was estimated to be an amplified current mediated by the concerted oxidation of Fc and the catechol moiety, which are in spatial proximity to each other in the CyD moiety.

As shown in Figure 5, *I*_pa1_ decreased with increasing boric acid concentration. The pH of the measurement solution was almost constant after boric acid addition (Figure S24). The decrease in *I*_pa1_ was not observed when 12 μL of ultrapure water was added (Figure S25).

### 2.4. Evaluation of Analytical Performance

#### 2.4.1. Limit of Detection

**Figure 6:**
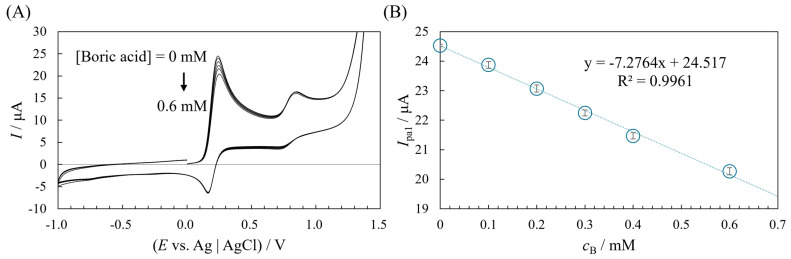
(**A**) CVs of solutions containing Fc/3,4-DHBA-*β*-CyD in 10% CH_3_OH/90% H_2_O (*v*/*v*) with boron concentrations ranging from 0 to 0.6 mM at pH 8.6 and r.t. [Fc] = 0.5 mM, [3,4-DHBA-*β*-CyD] = 1.5 mM, [NaCl] = 0.1 M, [phosphate] = 50 mM, and scan rate = 0.1 V s^−1^. WE: sGCDE (*ϕ* = 3.0 mm), RE: Ag|AgCl _3M NaCl aq._, CE: Pt coil. (**B**) Plot of *I*_pa1_ as a function of *c*_B_ (*n* = 3).

CV data were acquired at lower boron concentrations of 0 to 0.6 mM to calculate the limit of detection (LOD) (Figure 6A). *I*_pa1_ decreased with increasing concentration of boric acid (*c*_B_). A calibration curve was obtained by plotting *I*_pa1_ as a function of *c*_B_, and LOD was calculated from the slope of this relationship as 3*σ*_0_/slope based on a *σ*_0_ value of 0.036 obtained from three replicate trials (Figure 6B). This calculation gave an LOD of 0.16 mg B L^−1^ (14.9 μM).

#### 2.4.2. Analysis of Spiked Samples

To evaluate the practicality of this method based on Fc/3,4-DHBA-*β*-CyD, spike recovery tests were conducted using puddle water, river water, and tap water samples (Figures S26 and S27). The same response to boron was obtained for these samples as for ultrapure water, and the calibration curves showed good linearity (Table 1). Suitable recoveries were observed, indicating that this method is applicable to real samples.

#### 2.4.3. Selectivity for Boron

The selectivity of the present system based on Fc/3,4-DHBA-*β*-CyD for boric acid was investigated by comparing the decrease in *I*_pa1_ in the presence of 5.0 mM boron or other ions at a concentration of 5.0 mM (Figures S28–S31). The incorporation of Ba^2+^, Ca^2+^, Cd^2+^, K^+^, Li^+^, Mg^2+^, CH_3_COO^−^, ClO_4_^−^, F^−^, I^−^, NO_3_^−^, or SO_4_^2−^ into the reaction solution had no effect on *I*_pa1_. In contrast, *I*_pa1_ was decreased by the addition of Al^3+^, Cu^2+^, or Ni^2+^.

#### 2.4.4. Stability of 3,4-DHBA-*β*-CyD

**Figure 7:**
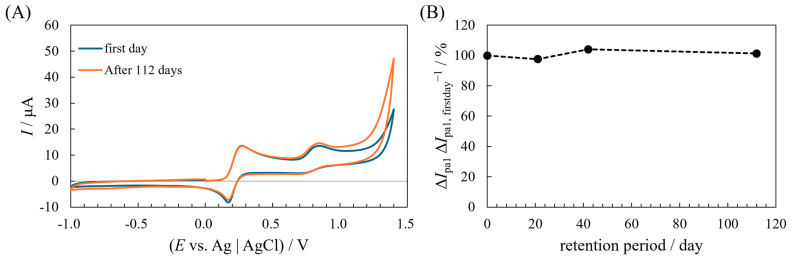
(**A**) The comparison of CVs on the first day (blue line) and after 112 days (orange line). Fc/3,4-DHBA-*β*-CyD in 10% CH_3_OH/90% H_2_O (*v*/*v*) at r.t. with boron (*c*_B_ = 5 mM) at pH 8.6. WE: sGCDE (*ϕ* = 3.0 mm), RE: Ag|AgCl _3M NaCl aq._, and CE: Pt coil. (**B**) The relationship between the retention period and the responsibility of Fc/3,4-DHBA-*β*-CyD to boron (*n* = 3). Δ*I*_pa1_ = (*I*_pa1 without boric acid_) − (*I*_pa1 with 5.0 mM of boric acid_), Δ*I*_pa1, firstday_ is the value of Δ*I*_pa1_ at the day solution prepared.

Ensuring reagent stability is crucial for all the analytical methods, so we evaluated the stability of 3,4-DHBA-*β*-CyD. We dissolved 3,4-DHBA-*β*-CyD in 50 mM aqueous phosphate buffer containing 0.1 M NaCl. The solution was purged with argon gas and stored in an amber container at 3 °C in a refrigerator (Figure S32). CV data were acquired from the mixtures of this solution with a solution of Fc in methanol both immediately after the preparation of the 3,4-DHBA-*β*-CyD solution and 112 days later (Figure S33). The response of the Fc/3,4-DHBA-*β*-CyD system to boron remained unchanged after 112 days (Figure 7), indicating that the reagent was stable, at least for this period.

## 3. Discussion

**Scheme 1:**
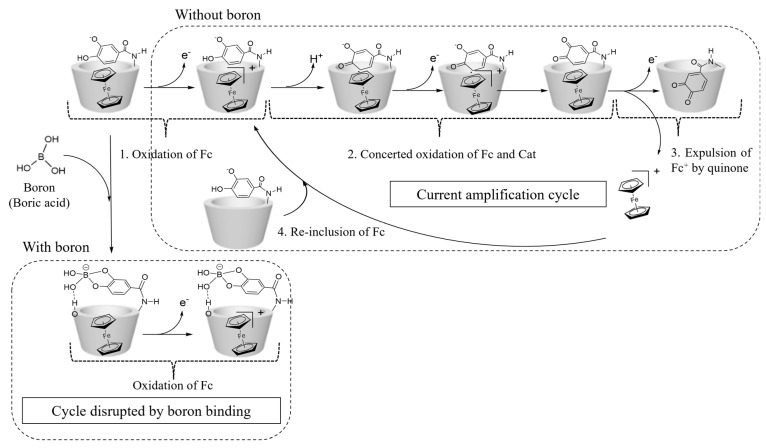
Plausible mechanism of boron detection in this study [48].

The current amplification in the voltammetry of catechol-functionalized *β*-CyD in the presence of ferrocene (Fc) is originated from an EC’ reaction that features a concerted oxidation through steric interactions involving the inclusion of Fc into the functionalized CyD (Scheme 1, [48]). The addition of boron decreases this amplified current because boric acid is bound to the catechol moiety, inhibiting the concerted oxidation.

M. A. Casulli et al. reported that structural changes in a 4-ferrocene-phenylboronic acid probe and *β*-CyD supramolecular complex for selective fructose recognition affected the electrochemical response to the target molecule [49]. Based on the structural electrochemical effects, we investigated the effects of the CyD functionalization site on boric acid detection. The amplified currents of inclusion complexes except Fc/2,5-DHBA-*β*-CyD were markedly decreased in the presence of boron. We presumed that this decrease in the amplified current was caused by the binding of boric acid to *cis*-diol, disrupting the amplification cycle [48]. The largest response was observed for Fc/3,4-DHBA-*β*-CyD (Figure 2H). Thus, Fc/3,4-DHBA-*β*-CyD was investigated further.

The optimum pH for the complexation between boric acid (acid dissociation constant p*K*_aB_ = 8.98) and a diol (acid dissociation constant p*K*_adiol_) can be calculated using the following equation: pH = (p*K*_aB_ + p*K*_adiol_)/2 [38,50,51]. In our previous report [48], the p*K*_a_ of Fc/3,4-DHBA-*β*-CyD (p*K*_aFc/3,4-DHBA-*β*-CyD_) was calculated to be 8.23. A p*K*_aB_ of 8.98 at 25 °C has been reported for boric acid [38,52]. Thus, the optimum pH for the reaction of Fc/3,4-DHBA-*β*-CyD with boric acid was estimated to be 8.6 ((p*K*_aFc/3,4-DHBA-*β*-CyD_ + p*K*_aB_)/2 = 8.6). This value was consistent with the results of the experiments (Figure 4).

As shown in Figure 5, *I*_pa1_ decreased with increasing boric acid concentration under optimal pH value (pH 8.6). The pH of the measurement solution was almost constant after boric acid addition (Figure S24). The decrease in *I*_pa1_ was not observed when 12 μL of ultrapure water was added (Figure S25). From these results, we estimated that the decrease in peak current after adding boric acid was caused by the interaction between boric acid and Fc/3,4-DHBA-*β*-CyD. In our previous report [48], we estimated that the current amplification was brought about by a concerted oxidation of the Cat moiety of 3,4-DHBA-*β*-CyD and Fc within the CyD moiety, which are in spatial proximity to each other and have the same oxidation potential. The binding of boron to the Cat moiety reduced the amplified current because it presumably interfered with Cat oxidation, preventing the concerted oxidation from occurring.

The binding constant for the interaction between Fc/3,4-DHBA-*β*-CyD and boric acid was estimated to be approximately 1500 M^−1^ on the basis of analysis of UV-vis spectra acquired from the aqueous solutions of Fc/3,4-DHBA-*β*-CyD at pH 8.6 with various boron concentrations (Figure S34). These data were analyzed using the MS-Excel 2021 program based on the Benesi–Hildebrand method [53,54]. The binding constant for the interaction between boric acid and a *cis*-diol structure is typically on the order of 10^2^ M^−1^ [27,28,39]; thus, the value obtained in the present study was relatively high. This discrepancy could be attributed to enhanced interactions between boric acid and the *β*-CyD structure via hydrogen bonding. It has been reported that boric acid and compounds with boric acid moieties undergo weak binding to hydroxyl groups at the 2 and 3 positions of CyD through hydrogen bonding [55].

From the result of Section 2.4.1, the LOD was calculated to be 0.16 mg B L^−1^ (14.9 μM), suggesting that this electrochemical method could detect boron at lower potential than previous study and sub-ppm levels, the same order of sensitivity as other methods (Table S2). Both differential pulse voltammetry (DPV) and square pulse voltammetry (SWV) are more sensitive than CV in general, so DPV (Figures S35 and S36; Tables S3 and S4) and SWV (Figures S37 and S38; Tables S5 and S6) were also performed at boron concentrations ranging from 0 to 0.6 mM. Both techniques demonstrated that *I*_pa1_ decreased linearly with increasing *c*_B_. However, the lowest LOD value was calculated from CV data obtained at a scan rate of 100 mV/s, meaning that this scan rate gave the highest sensitivity (Figures S39 and S40; Tables S7 and S8)

In the competitive experiments, *I*_pa1_ was affected by the addition of Al^3+^, Cu^2+^, or Ni^2+^. This was attributed to the interactions (like chelate coordination) of these ions with the *cis*-diol moiety of Fc/3,4-DHBA-*β*-CyD, which inhibited the binding of the *cis*-diol moiety with boric acid.

Unlike other CMs based on simple 1:1 binding, our method is based on changes in electrochemical amplifying response using supramolecular interactions. The binding of a single molecule of boric acid produces an effect likened to that of multiple molecules, realizing relatively highly sensitive boron detection. Our boron detection method is rapid and straightforward, utilizing relatively stable reagents, not hazardous material.

## 4. Materials and Methods

### 4.1. Apparatus

All the filtration processes for real sample measurements and the synthesis of *β*-CyDs combined with benzoic acid derivatives were performed using membranes having 450 nm pores. Both the ^1^H and ^13^C nuclear magnetic resonance (NMR) spectra were acquired using a JNM-ECX500 spectrometer (JEOL Ltd., Tokyo, Japan) at 298 K. High-resolution electrospray ionization mass spectrometry (HR-ESI-MS) analyses were carried out with a JEOL Accu-TOF JMS T100LC instrument (JEOL Ltd., Tokyo, Japan). The pH values were determined using an F-72 pH meter (HORIBA Ltd., Kyoto, Japan). The electrochemical measurements were performed with an Autolab PGSTST128N potentiostat (Metrohm AG, Switzerland) in conjunction with a Nova 2.1.5 software package (Metrohm Autolab BV, Netherlands). These measurements employed a 3 mm diameter glassy carbon electrode (BAS Inc., Tokyo, Japan) as the working electrode, an Ag|AgCl _3M NaClaq_ reference electrode (BAS Inc., Tokyo, Japan), and a 23 mm platinum wire as the counter electrode (BAS Inc., Tokyo, Japan). Ultraviolet-visible (UV-vis) absorption spectra were acquired with a V-570 spectrophotometer (Jasco Inc., Tokyo, Japan) using a 1.0 cm quartz cell.

### 4.2. Reagents

For CyD functionalization, acetone (Kanto Chemical Co., Inc., Tokyo, Japan), (2A*S*,3A*S*)-3A-amino-3A-deoxy-*β*-cyclodextrin (3-NH_2_-*β*-CyD, Tokyo Chemical Industry Co., Ltd., Tokyo, Japan), *N*,*N*-dimethylformamide (DMF, FUJIFILM Wako Pure Chemical, Osaka, Japan), *N*,*N*′-dicyclohexylcarbodiimide (DCC, Tokyo Chemical Industry Co., Ltd., Tokyo, Japan), and 1-hydroxybenzotriazole monohydrate (HOBt·H_2_O, Tokyo Chemical Industry Co., Ltd., Tokyo, Japan) were used. Benzoic acid (BA), 2-hydroxybenzoic acid (2-HBA), 3-hydroxybenzoic acid (3-HBA), 4-hydroxybenzoic acid (4-HBA), 2,5-dihydroxybenzoic acid (2,5-DHBA), 2,6-dihydroxybenzoic acid (2,6-DHBA), 3,4-dihydroxybenzoic acid (3,4-DHBA), 3,5-dihydroxybenzoic acid (3,5-DHBA), gallic acid hydrate (3,4,5-THBA), and caffeic acid (3,4-DHCA) were purchased from Tokyo Chemical Industry Co., Ltd. (Tokyo, Japan). For NMR measurement, methanol-d4 (99.8% D, Kanto Chemical Co., Inc., Tokyo, Japan), deuterium oxide (99.8% D, Kanto Chemical Co., Inc., Tokyo, Japan), and acetone (infinity pure, FUJIFILM Wako Pure Chemical, Osaka, Japan) were used. For electrochemical measurement, ferrocene (Tokyo Chemical Industry Co., Ltd., Tokyo, Japan), methanol (FUJIFILM Wako Pure Chemical, Osaka, Japan), sodium chloride (NaCl, FUJIFILM Wako Pure Chemical, Osaka Japan), sodium dihydrogen phosphate anhydrous (FUJIFILM Wako Pure Chemical, Osaka Japan), disodium hydrogen phosphate (FUJIFILM Wako Pure Chemical, Osaka Japan), sodium hydroxide (NaOH, FUJIFILM Wako Pure Chemical, Osaka Japan), hydrochloric acid (HCl, FUJIFILM Wako Pure Chemical, Osaka Japan), and boric acid (Kanto Chemical Co., Inc., Tokyo, Japan) were used. For the selectivity tests, copper(II) chloride dihydrate (CuCl_2_·2H_2_O, Kanto Chemical Co., Inc., Tokyo, Japan), sodium iodide (NaI, Kanto Chemical Co., Inc., Tokyo, Japan), and sodium fluoride (NaF, Sigma-Aldrich, Co. LLC., Tokyo, Japan) were used. Aluminum(III) chloride hexahydrate (AlCl_3_·6H_2_O), barium chloride dihydrate (BaCl_2_·2H_2_O), calcium chloride anhydrous (CaCl_2_), cadmium chloride (CdCl_2_), potassium chloride (KCl), lithium chloride (LiCl), magnesium chloride hexahydrate (MgCl_2_·6H_2_O), nickel(II) chloride hexahydrate (NiCl_2_·6H_2_O, 99.9%), sodium acetate (CH_3_COONa), sodium perchlorate monohydrate (NaClO_4_·H_2_O), sodium sulfate (Na_2_SO_4_), and sodium nitrate (NaNO_3_) were purchased from FUJIFILM Wako Pure Chemical (Osaka, Japan).

All the reagents mentioned above were used as received without further purification. Water was doubly filtered and deionized using an Elix water purification system and a Milli-Q water system (Elix Essential Advantage and Milli-Q Advantage, Merck Millipore, MA, USA) prior to use.

### 4.3. Synthesis of Functionalized CyDs

Functionalized CyDs were synthesized and identified as in previous studies [48]. See below and SI for identification data.

Analytical data for 2-HBA-*β*-CyD are as follows: Yield: 78.2%; HR-MS (ESI-) (calcd for C_49_H_74_NO_36_ ([M-H])): *m*^+^/*z* = 1252.39281 (1252.39905); ^1^H NMR (500 MHz, D_2_O): δ 7.76–7.80 (d, 1H), 7.41–7.47 (t, 1H), 6.97–7.04 (t, 1H), 6.82–6.87 (d, 1H), 4.78–5.05 (m, 7H), 3.06–4.03 (m, 42H); UV-vis (H_2_O at pH 5.8): *λ*_max_/nm (log_10_(*ε*/mol^−1^ dm^3^ cm^−1^)) = 236.5 (3.94), 296 (3.54).

Analytical data for 3-HBA-*β*-CyD are as follows: Yield: 68.6%; HR-MS (ESI-) (calcd for C_49_H_74_NO_36_ ([M-H])): *m*^+^/*z* = 1252.40611 (1252.39905); ^1^H NMR (500 MHz, D_2_O): δ 7.28–7.34 (t, 1H), 7.10–7.16 (d, 1H), 6.98–7.03 (d and s, 2H), 4.77–5.01 (m, 7H), 3.26–4.01 (m, 42H); UV-vis (H_2_O at pH 7.0): *λ*_max_/nm (log_10_(*ε*/mol^−1^ dm^3^ cm^−1^)) = 291.5 (3.38).

Analytical data for 2,5-DHBA-*β*-CyD are as follows: Yield: 40.5%; HR-MS (ESI-) (calcd for C_49_H_74_NO_37_ ([M-H])): *m*^+^/*z* = 1268.39656 (1268.39397); ^1^H NMR (500 MHz, D_2_O): δ 7.28–7.32 (s, 1H), 6.98–7.03 (d, 1H), 6.71–6.75 (d, 1H), 4.85–5.05 (m, 7H), 3.35–4.05 (m, 42H); ^13^C NMR (125 MHz, D_2_O + 0.5% acetone): *δ* 121.43, 102.33, 102.09, 101.88, 101.60, 101.49, 100.40, 81.13, 80.77, 73.33, 73.19, 73.08, 72.63, 72.27, 72.19, 72.16, 72.06, 71.99, 71.74, 71.65, 60.64, 60.45, 60.33, 60.24, 60.10, 50.97; UV-vis (H_2_O at pH 5.9): *λ*_max_/nm (log_10_(*ε*/mol^−1^ dm^3^ cm^−1^)) = 322.0 (3.60); elemental analysis (calcd for C_49_H_75_NO_37_): C 46.10 (46.34)%, H 5.96 (5.95)%, and N 1.19 (1.10)%.

Analytical data for 2,6-DHBA-*β*-CyD are as follows: Yield: 34.7%; HR-MS (ESI-) (calcd for C_49_H_74_NO_37_([M-H])): *m*^+^/*z* = 1268.48103 (1268.39397); ^1^H NMR (500 MHz, D_2_O): δ 7.09–7.13 (t, 1H), 6.24–6.28 (d, 2H), 4.85–5.01 (m, 7H), 3.2–3.96 (m, 42H), 3.35–4.05 (m, 42H); UV-vis (H_2_O at pH 7.0): *λ*_max_/nm (log_10_(*ε*/mol^−1^ dm^3^ cm^−1^)) = 248.0 (3.82), 308.0 (3.45).

Analytical data for 3,4,5-THBA-*β*-CyD are as follows: Yield: 96.9%; HR-MS (ESI-) (calcd for C_49_H_75_NO_38_): m^+^/z = 1285.40405 (1285.39670); ^1^H NMR (500 MHz, D_2_O): δ 6.77–6.83 (s,2H), 4.84–5.07 (m, 7H), 3.37–4.07 (m, 42H); ^13^C NMR (125 MHz, D_2_O + 0.5% acetone): δ 170.35, 145.13, 137.16, 125.06, 124.93, 124.83, 124.75, 117.67, 111.30, 102.10, 102.05, 101.86, 101.81, 101.68, 101.63, 101.40, 101.15, 81.34, 81.15, 81.03, 80.92, 73.21, 73.13, 72.05, 71.99, 71.88, 71.80, 60.39, 60.30, 60.20, 51.49; UV-vis (H_2_O at pH 6.5): *λ*_max_/nm (log_10_(*ε*/mol^−1^ dm^3^ cm^−1^)) = 268.0 (3.81).

Analytical data for 3,4-DHCA-*β*-CyD are as follows: Yield: 72.6%; MS (ESI-) (calcd for C_51_H_77_NO_37_): *m*^+^/*z* = 1295 (1295); ^1^H NMR (500 MHz, D_2_O): δ 7.25–7.33 (d, 1H), 6.71–6.95 (d and s, 3H), 5.31–5.39 (d, 1H), 4.85–5.00 (m, 7H), 3.20–4.00 (m, 42H).

### 4.4. Electrochemical Measurements

All the experiments on electrochemical measurements were performed with a three-electrode system described in Section 4.1. Solutions for the electrochemical measurements were prepared by adding a methanol solution containing Fc to an aqueous solution comprising phosphate buffer (50 mM), NaCl (0.1 M), and a functionalized *β*-CyD. The final concentrations of the functionalized *β*-CyD and Fc were 1.5 and 0.5 mM, respectively, because we found that the amplified current was maximum at these concentrations in our previous work [48]. The pH of the resulting solution was adjusted by adding aqueous solutions of either NaOH or HCl. The samples of puddle water, river water, and tap water were passed through a membrane filter (JHWP) under a vacuum to remove particles and other contaminants before analysis. In the experiments investigating selectivity for boron, aqueous solutions of AlCl_3_·6H_2_O, BaCl_2_·2H_2_O, CaCl_2_, CdCl_2_, CuCl_2_·2H_2_O, KCl, LiCl, MgCl_2_·6H_2_O, NiCl_2_·6H_2_O, CH_3_COONa, NaClO_4_, NaF, NaI, NaNO_3_, or Na_2_SO_4_ were prepared and added to the test solution to provide a final concentration of 5.0 mM. CV data were acquired before and after adding boron to these samples in the form of an aqueous solution of boric acid. CV analyses were performed over a potential window between −1.0 and 1.4, 1.2, or 1.0 V at a scan rate of 0.1, 0.2, or 0.4 V s^−1^ with a quiet time of 5 s at room temperature. All the solutions were purged with argon gas before the analysis.

DPV data were acquired over a potential window between 0 and 1.0 V with a pulse step of 5 mV; a modulation amplitude (pulse amplitude) of 10, 20, 30, or 50 mV; a modulation time (pulse width) of 60 ms; an interval time (pulse period) of 0.2 s; and a quiet time of 5 s at room temperature. Each solution was purged with argon gas before each test.

SWV data were acquired over a potential window between 0 and 1.0 V with a pulse step of 4 mV; a modulation amplitude (pulse amplitude) of 25 or 50 mV; a frequency of 25, 50, or 90 Hz; and a quiet time of 5 s at room temperature. Each solution was purged with argon gas before each test.

### 4.5. UV-Vis Spectroscopy

A methanol solution containing Fc was added to 3,4-DHBA-*β*-CyD aqueous solution to give a final concentration of [3,4-DHBA-*β*-CyD] = 60 μM and [Fc] = 20 μM. In the experiment to determine the binding constant for the interaction between Fc/3,4-DHBA-*β*-CyD and boric acid (Figure S10), the UV-vis measurements of this solution (10% CH_3_OH/90% H_2_O (*v*/*v*)) at various boron concentrations were performed.

### 4.6. Data Analysis

Analysis of the results of each voltammetry and UV-vis spectroscopy was performed by the MS-Excel 2021 program. The error bars are the standard deviation values of three measurements. The average value of three measurements was used for the plots.

## 5. Conclusions

We proposed a boron detection method based on current amplification through 1:1 binding between boron and a Fc/catechol-functionalized *β*-CyD inclusion complex. The binding of a single boron molecule to the catechol moiety disrupted the current amplification cycle. The detection sensitivity was improved compared to our previously reported method that employs simple binding between the *cis*-diol moiety of a ruthenium complex and boric acid [39].

The response of the supramolecular complexes of Fc and *β*-CyDs having various functionalization sites was investigated to determine the optimum structure for boron detection. We found that Fc/3,4-DHBA-*β*-CyD showed the largest electrochemical response to boric acid. Both p*K*_a_ calculations and electrochemical measurements confirmed that Fc/3,4-DHBA-*β*-CyD showed the largest response to boron at pH 8.6. CV, DPV, and SWV with various parameters revealed that the lowest LOD was 0.16 mg B L^−1^ at a scan rate of 0.1 V s^−1^. Our method was used for detecting boron in the puddle water, tap water, and river water samples. We found that the 3,4-DHBA-*β*-CyD reagent remained stable for at least 112 days. The selectivity tests showed that our method could detect boron in the presence of various anions or cations. Our method, which is based on current amplification through supramolecular interaction and does not use hazardous reagents, achieved straightforward and rapid boron detection at a lower potential than the tiron method [37] and with similar sensitivity to most previous reports (Table S5). Our method has linearity for detecting boron at a range of 0.2–6.5 mg B L^−1^ (Figure 6). Therefore, it has the potential to be applicable for monitoring boron concentrations in drinking water and wastewater. Cyclodextrin-based reagents can be used for various applications, such as chemically modified electrodes and nanogels for further improvement.

## Figures and Tables

**Figure 1:**
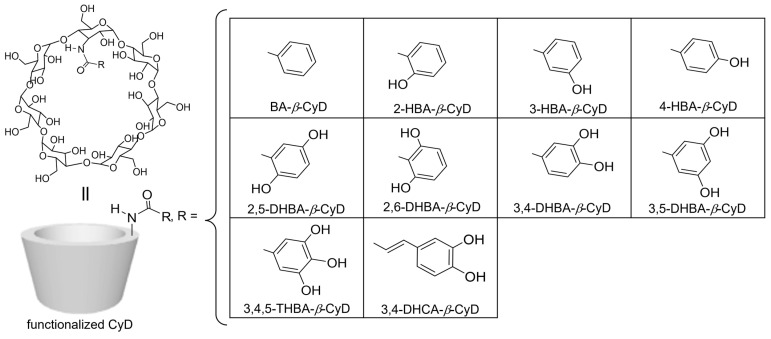
Proposed structure of supramolecular complexes between Fc and *β*-CyD functionalized with benzoic acid derivatives in this study.

**Table 1:** Results of boron spike recovery tests using real samples.

	Added/mM	Found/mM	Recovery/%	RSD/%	*R* ^2^
Puddle water	0.60	0.56	93.3	1.36	0.9874
River water	0.60	0.68	113.2	0.81	0.9959
Tap water	0.60	0.59	98.6	1.93	0.9631

## Data Availability

Data will be made available upon request.

## Supplementary Materials

The following supporting information can be downloaded at https://www.mdpi.com/article/10.3390/ijms26094432/s1.
