# Supplementary material for: Electrochemical Boron Detection with Ferrocene and Catechol-Functionalized Cyclodextrin Inclusion Complex
Source: Int J Mol Sci. 2025 May 7;26(9):4432. doi: 10.3390/ijms26094432 (PMC12072688; doi:10.3390/ijms26094432)
Supplement: Supplementary file 1 [file ijms-26-04432-s001.zip › ijms-3601226-20250508_Supplementary Information v02 nomark.pdf]

Supplementary Information

Electrochemical Boron Detection with Ferrocene and Catechol-Functionalized Cyclodextrin Inclusion Complex

Authors

Kai Sato[1], Hiroshi Kimoto[2], and Takeshi Hashimoto[1,*]

[1] Department of Materials and Life Sciences, Faculty of Science and Technology, Sophia University, 7-1 Kioi-cho, Chiyoda-ku, Tokyo 102-8554, Japan

[2] Technical Development Division, Nomura Micro Science Co., Ltd., Atsugi, Kanagawa 243-0021, Japan

Table of Contents

1. **Preparation and identification of functionalized CyD · · · · · · S3**
2. **CVs and DPVs of functionalized CyD and Fc/functionalized CyD · S16**
3. **Confirmation of inclusion complex formation · · · · · · · · · · S26**
4. **The pH dependence of the response to boron · · · · · · · · · · S27**
5. **Effect of adding boric acid aqueous solution on pH · · · · · · S28**
6. **CV stability of Fc/3,4-DHBA-*β*-CyD · · · · · · · · · · · · · · S29**
7. **Spike test recovery with puddle water, river water, and tap water · S30**
8. **Effects of coexisting ions · · · · · · · · · · · · · · · · · · · S32**
9. **Stability of reagents · S38**
10. **The comparison with the previously reported boron determination methods · S40**
11. **Binding constant for the interaction between Fc/3,4-DHBA-*β*-CyD and boric acid · S41**
12. **DPV response to boron · S42**
13. **SWV response to boron · S44**
14. **Optimization of CV scan rate · · · · · · · · · · · · · · · · · S47**

1. Preparation and identification of functionalized CyDs

1-1. Preparation and identification of 2-HBA-β-CyD

2-HBA-β-CyD was synthesized as shown in Scheme S1. Identification data of 2-HBA-β-CyD were shown in Figures. S1-S3.

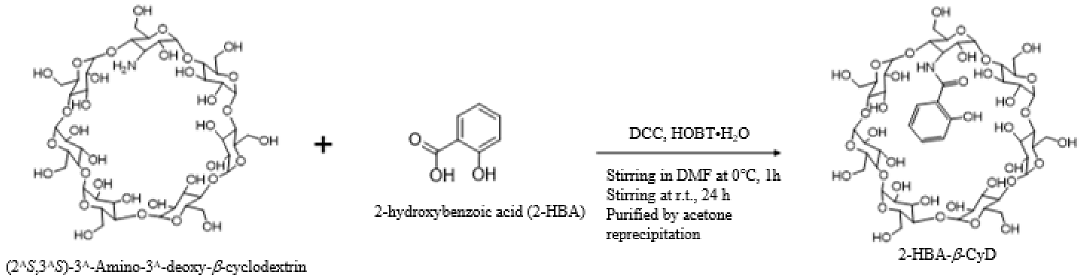

Scheme S1. Synthesis of 2-HBA-β-CyD.

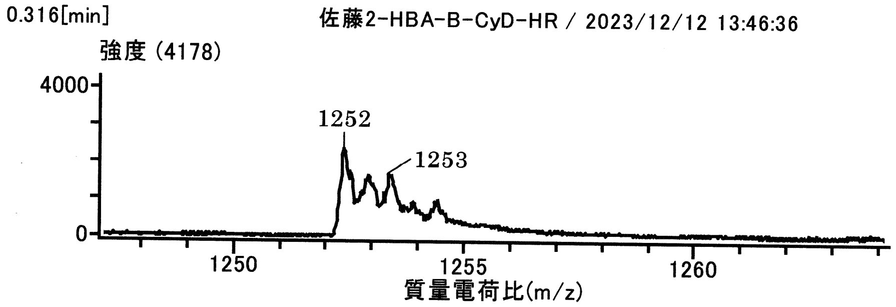

Figure. S1. HR-MS (ESI-) spectrum of 2-HBA-β-CyD.

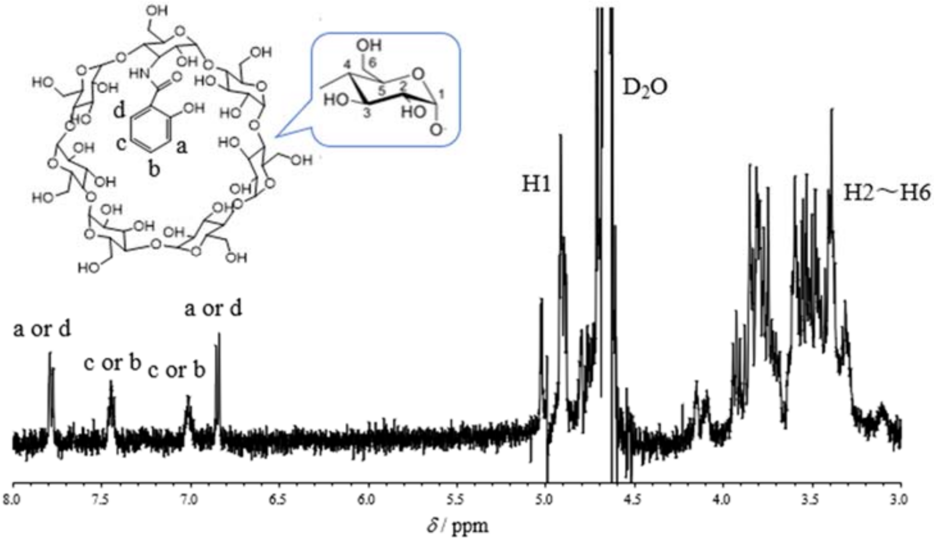

Figure. S2. ^{1}H NMR spectrum of 2-HBA-β-CyD in D_2O at 298 K, 500 MHz, 16 scans.

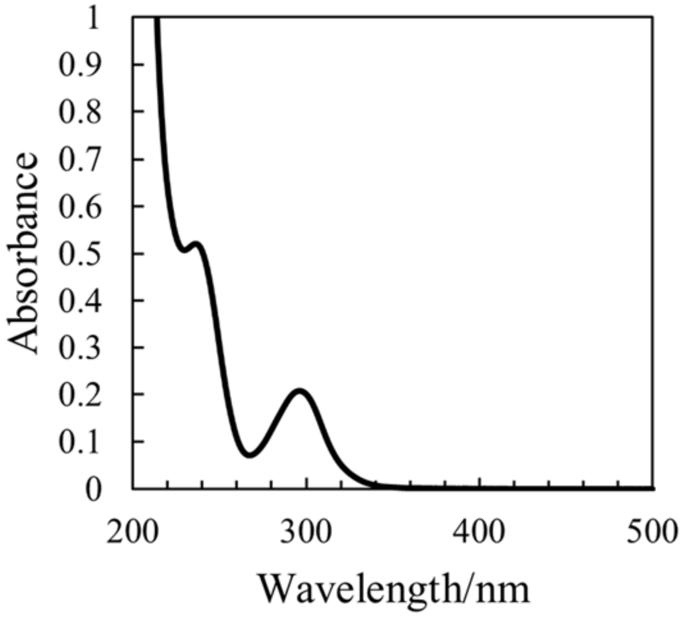

Figure. S3. UV-vis absorption spectrum of 2-HBA-β-CyD in H_2O at 25 °C. [2-HBA-β-CyD] = 60 μM.

1-2. Preparation and identification of 3-HBA-β-CyD

3-HBA-β-CyD was synthesized as shown in Scheme S2. Identification data of 3-HBA-β-CyD were shown in Figures. S4-S6.

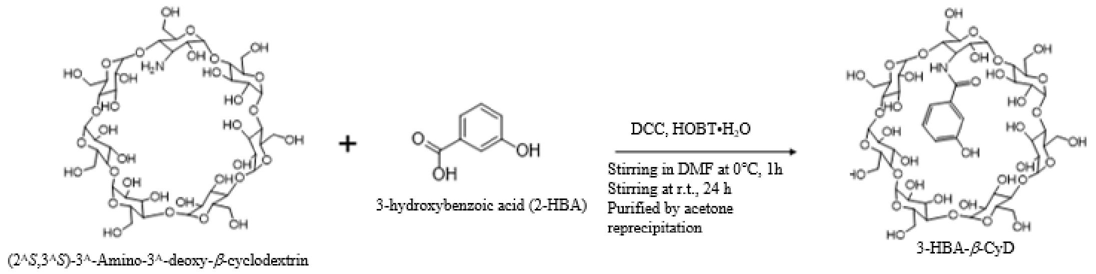

Scheme S2. Synthesis of 3-HBA-β-CyD.

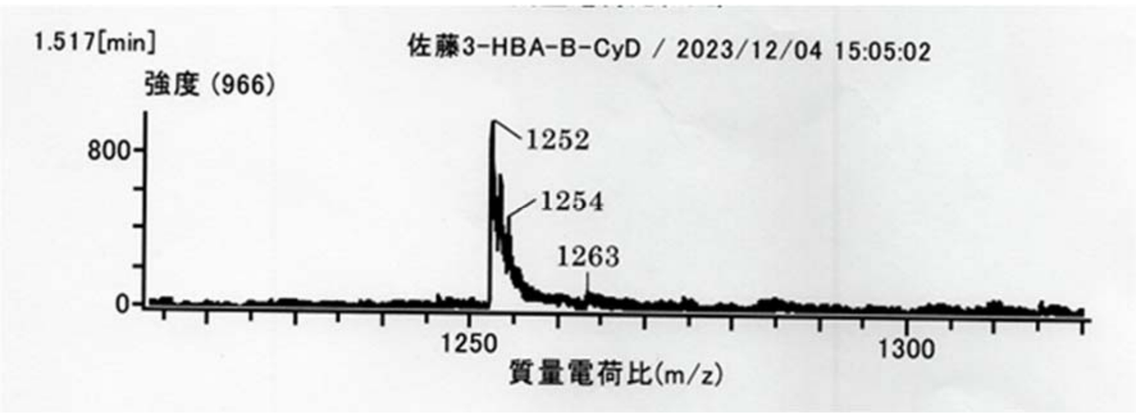

Figure. S4. HR-MS (ESI-) spectrum of 3-HBA-β-CyD.

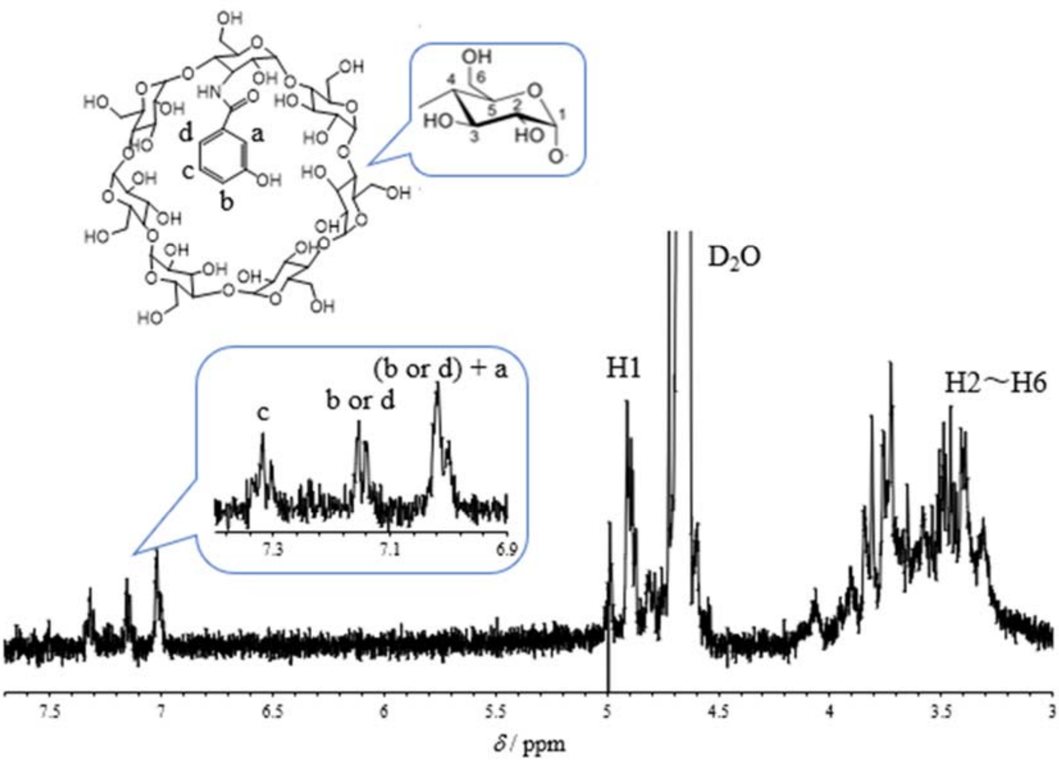

Figure. S5. ^{1}H NMR spectrum of 3-HBA-β-CyD in D_2O at 298 K, 500 MHz, 16 scans.

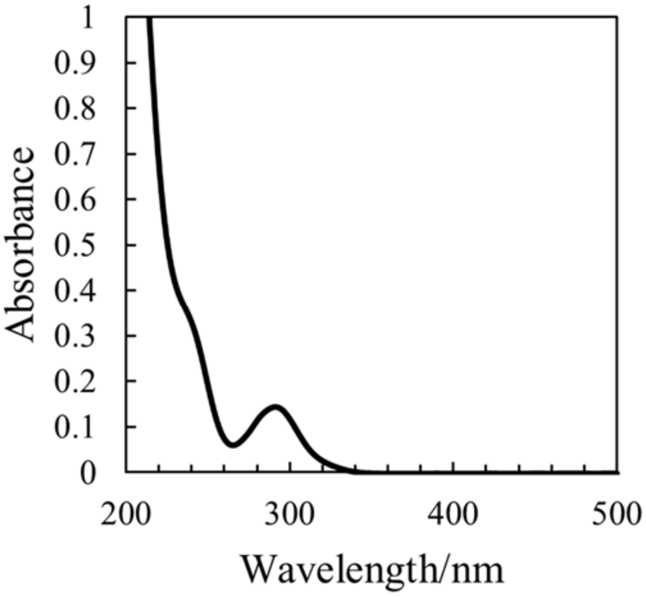

Figure. S6. UV-vis absorption spectrum of 3-HBA-β-CyD in H_2O at 25 °C. [3-HBA-β-CyD] = 60 μM.

1-3. Preparation and identification of 2,5-DHBA-β-CyD

2,5-DHBA-β-CyD was synthesized as shown in Scheme S3. Identification data of 2,5-DHBA-β-CyD were shown in Figures. S7-S10.

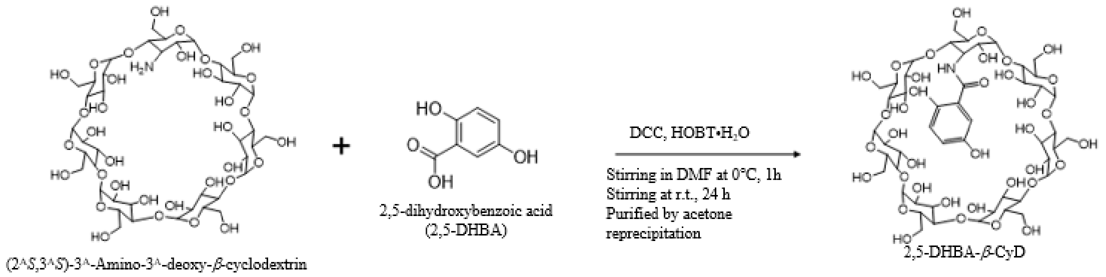

Scheme S3. Synthesis of 2,5-DHBA-β-CyD.

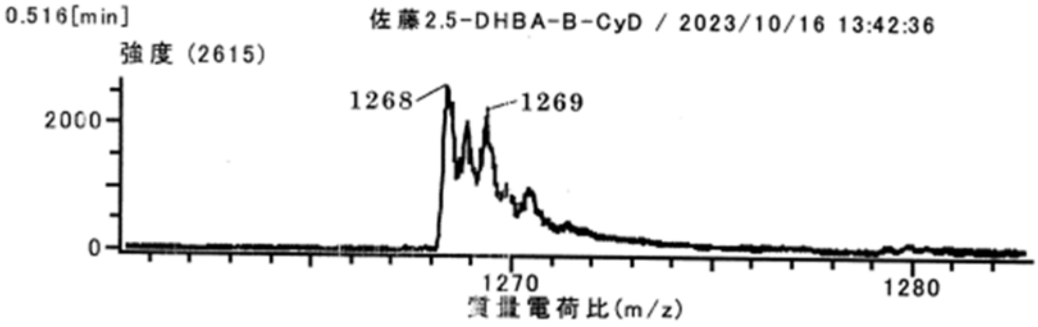

Figure. S7. HR-MS (ESI-) spectrum of 2,5-DHBA-β-CyD.

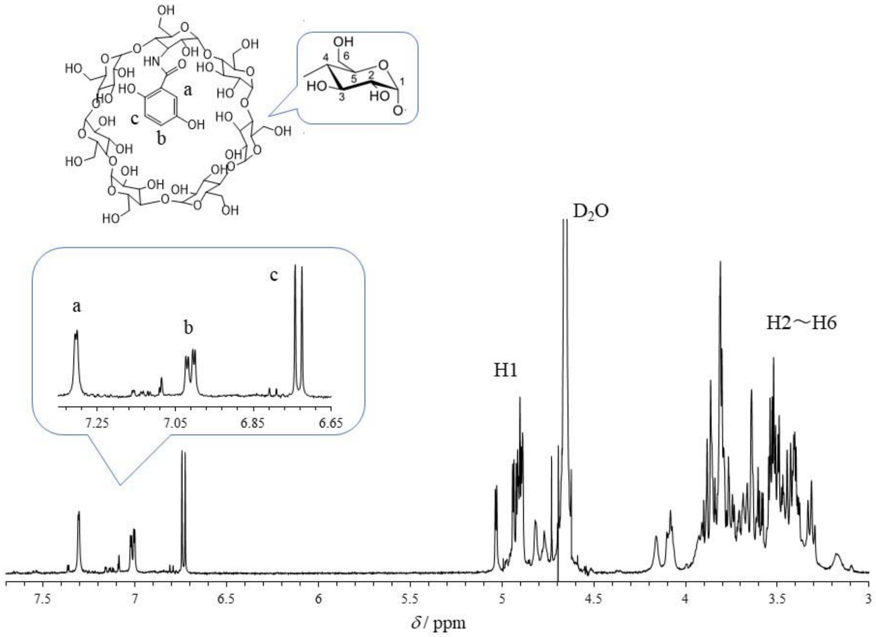

Figure. S8. ^{1}H NMR spectrum of 2,5-DHBA-β-CyD in D_2O at 298 K, 500 MHz, 16 scans.

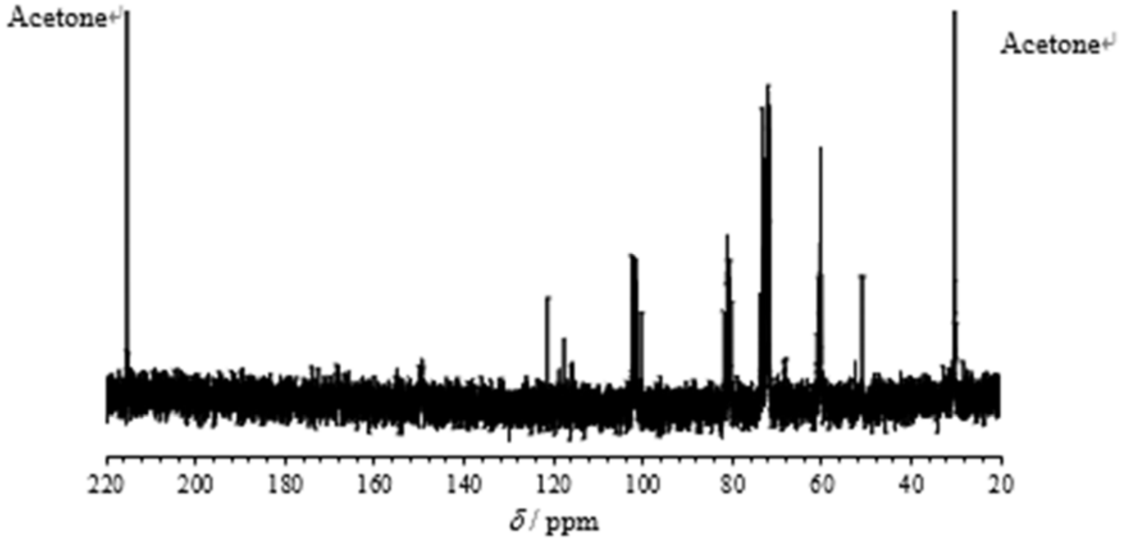

Figure. S9. ^{13}C NMR spectrum of 2,5-DHBA-β-CyD in 0.5 % acetone/99.5% D_2O (v/v) at 298 K, 125 MHz, 35000 scans, [2,5-DHBA-β-CyD] = 4.5 mM.

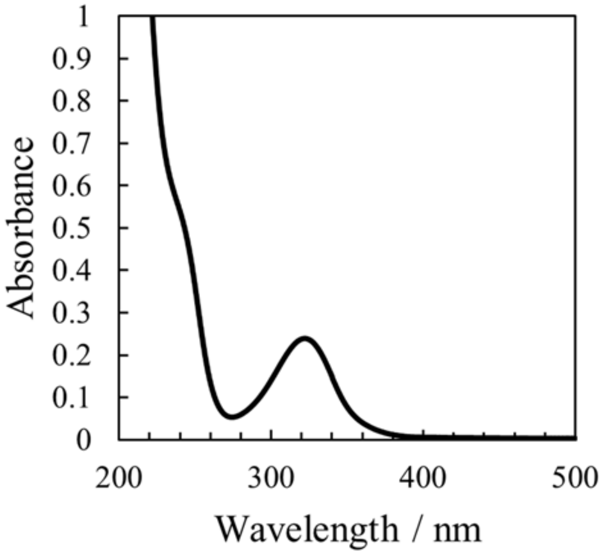

Figure. S10. UV-vis absorption spectrum of 2,5-DHBA-*β*-CyD in H_2O at 25 °C. [2,5-DHBA-*β*-CyD] = 60 μM.

1-4. Preparation and identification of 2,6-HBA-*β*-CyD

2,6-DHBA-*β*-CyD was synthesized as shown in Scheme S4. Identification data of 2,6-DHBA-*β*-CyD were shown in Figures. S11-S13.

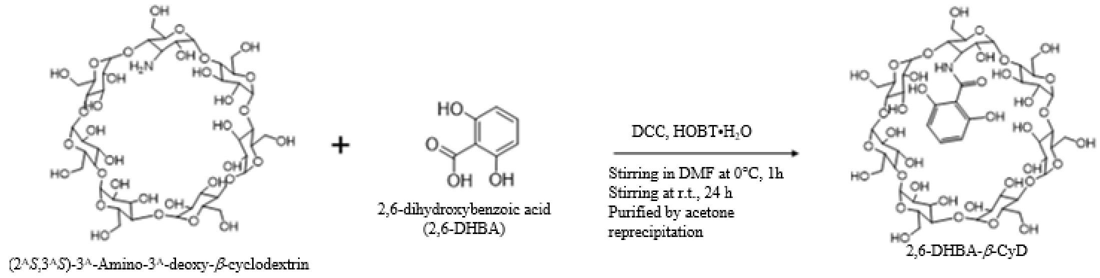

Scheme S4. Synthesis of 2,6-DHBA-*β*-CyD.

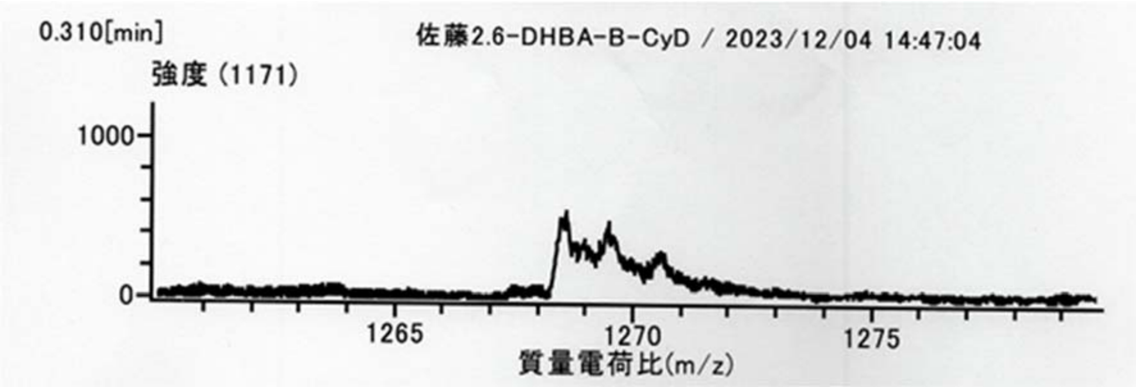

Figure. S11. HR-MS (ESI-) spectrum of 2,6-DHBA-*β*-CyD.

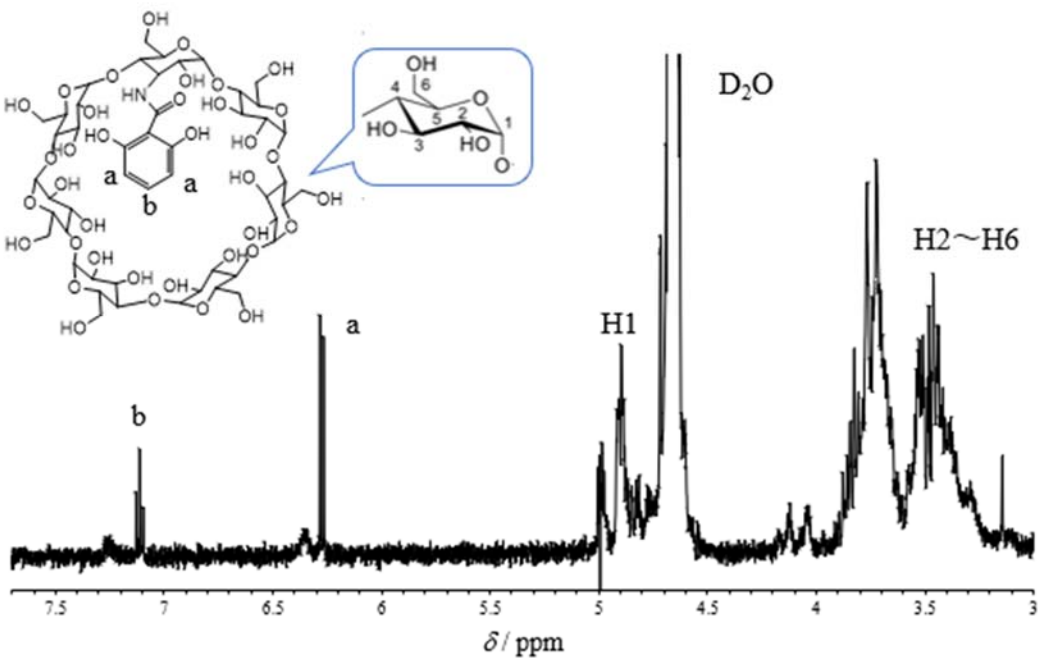

Figure. S12. ^{1}H NMR spectrum of 2,6-DHBA-β-CyD in D_2O at 298 K, 500 MHz, 16 scans.

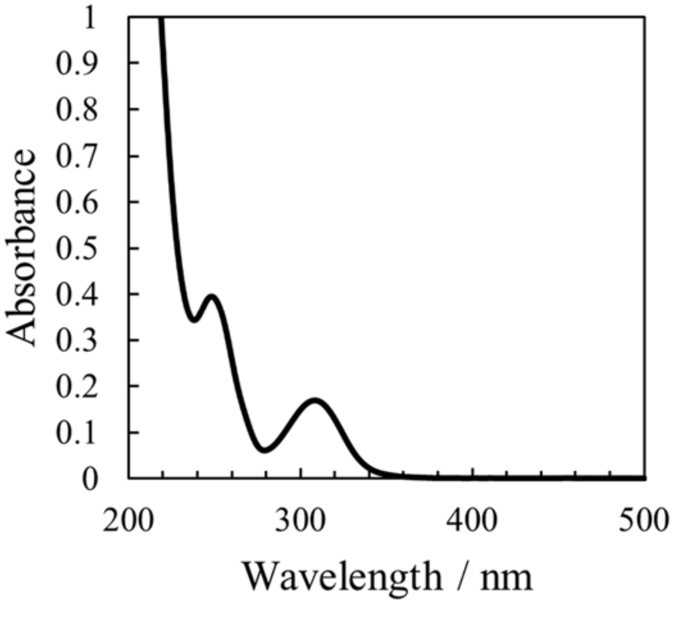

Figure. S13. UV-vis absorption spectrum of 2,6-DHBA-β-CyD in H_2O at 25 °C. [2,6-DHBA-β-CyD] = 60 μM.

1-5. Preparation and identification of 3,4,5-THBA-β-CyD

3,4,5-THBA-β-CyD was synthesized as shown in Scheme S5. Identification data of 3,4,5-THBA-β-CyD were shown in Figure. S14-S17.

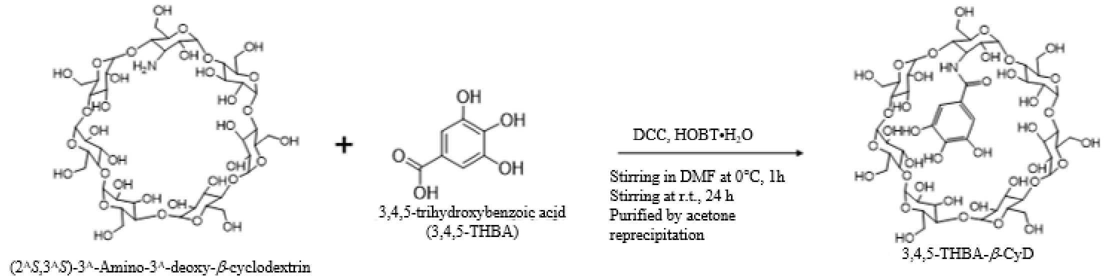

Scheme S5. Synthesis of 3,4,5-THBA-β-CyD.

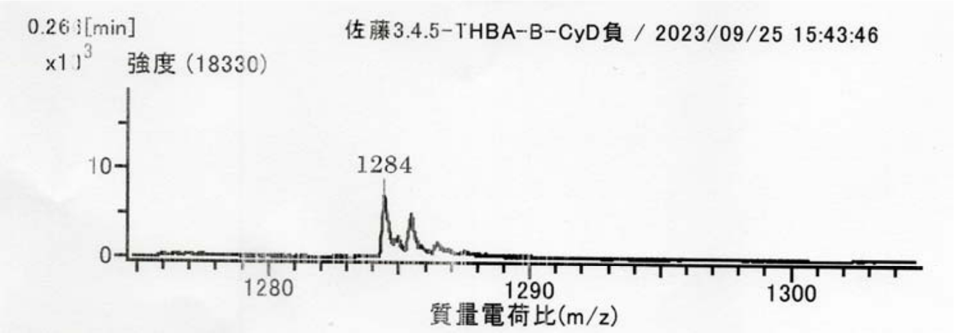

Figure. S14. HR-MS (ESI-) spectrum of 3,4,5-THBA-β-CyD.

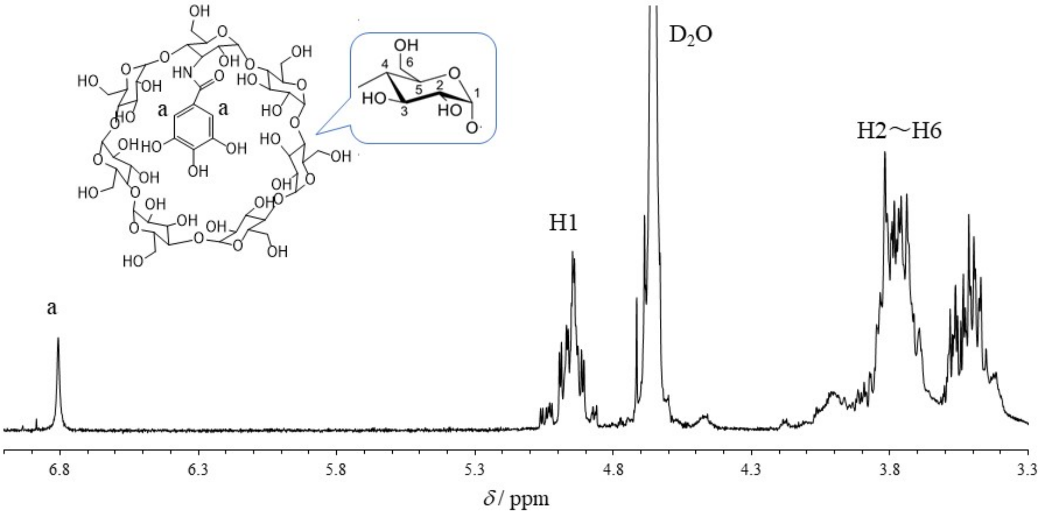

Figure. S15. ^{1}H NMR spectrum of 3,4,5-THBA-β-CyD in D_2O at 298 K, 500 MHz, 16 scans.

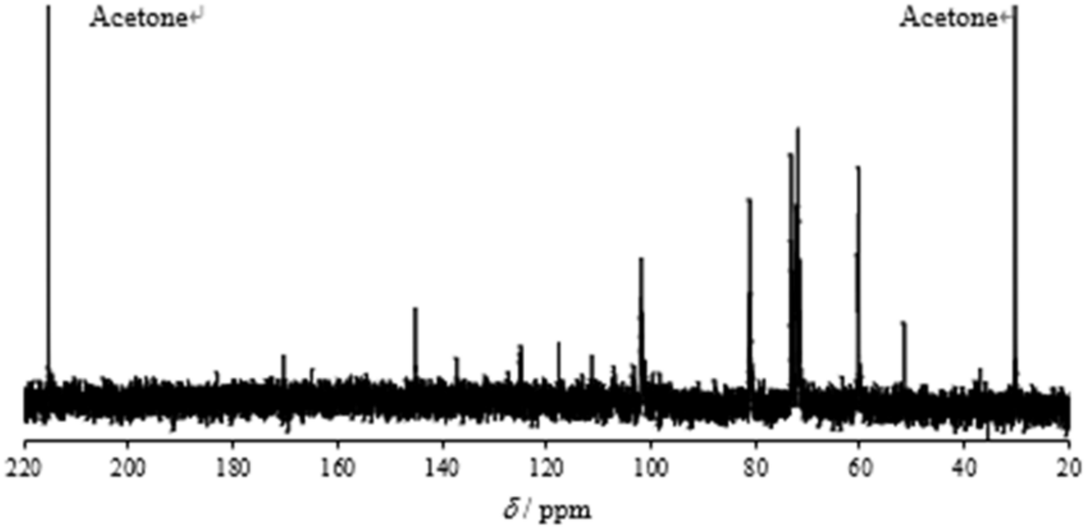

Figure. S16. ^{13}C NMR spectrum of 3,4,5-THBA-*β*-CyD in 0.5 % acetone/99.5% D_2O (v/v) at 298 K, 125 MHz, 35000 scans, [3,4,5-THBA-*β*-CyD] = 4.5 mM.

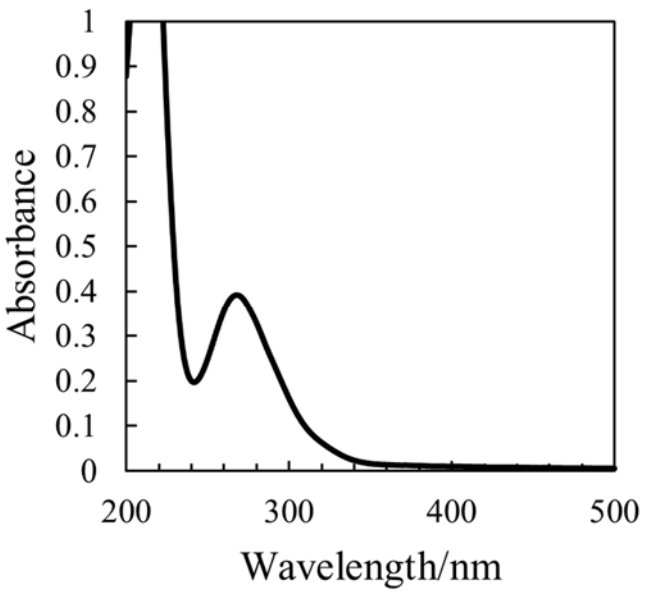

Figure. S17. UV-vis absorption spectrum of 3,4,5-THBA-*β*-CyD in H_2O at 25 °C. [3,4,5-THBA-*β*-CyD] = 60 μM.

1-6. Preparation and identification of 3,4-DHCA-β-CyD

3,4-DHCA-β-CyD was synthesized as shown in Scheme S6. Identification data of 3,4-DHCA-β-CyD were shown in Figures. S18-S19.

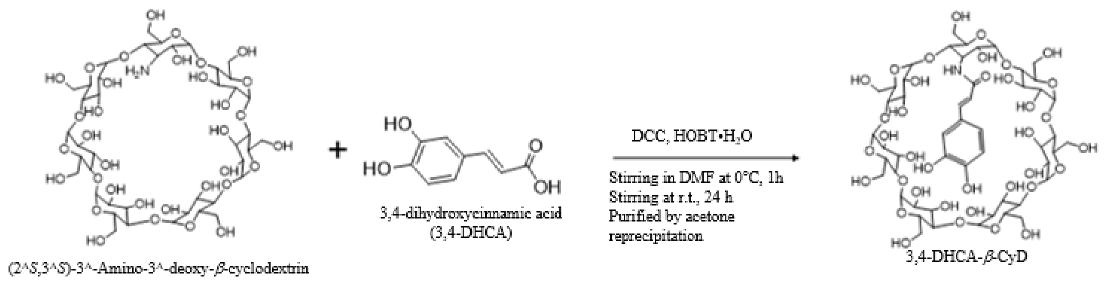

Scheme S6. Synthesis of 3,4-DHCA-β-CyD.

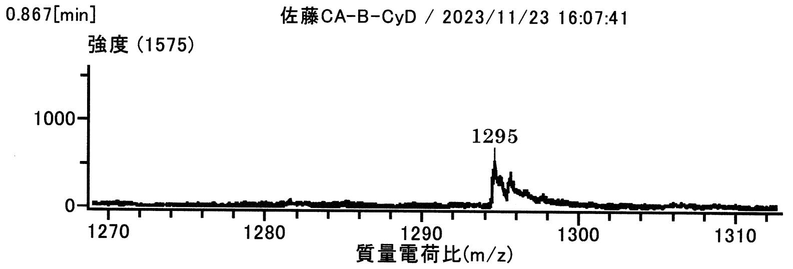

Figure. S18. HR-MS (ESI-) spectrum of 3,4-DHCA-β-CyD.

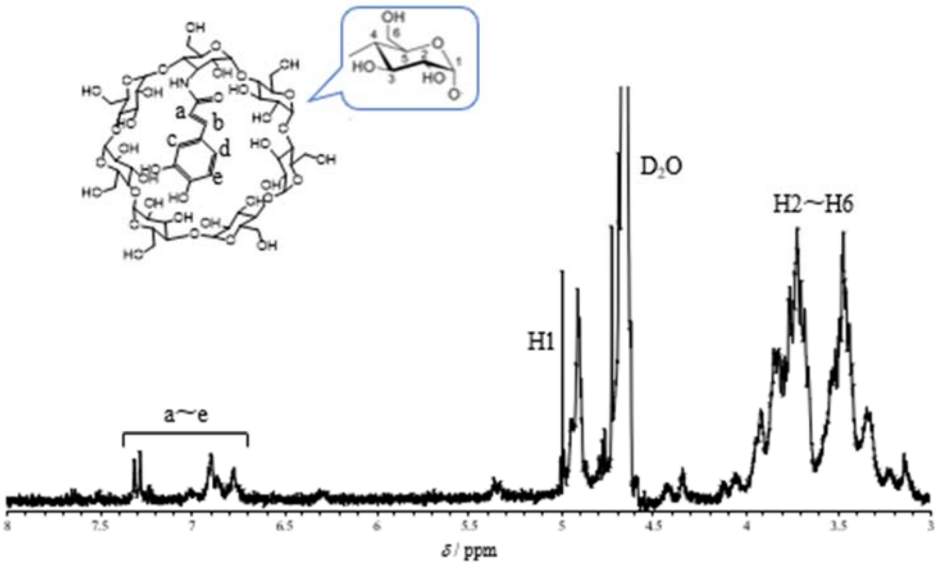

Figure. S19. ^{1}H NMR spectrum of 3,4-DHCA-β-CyD in D_2O at 298 K, 500 MHz, 16 scans.

1-7. The amount of reagents for the synthesis and yields

Table S1 The synthesis of functionalized β-CyD

Probe	Chemical Formula	Amount of reagent for synthesis							Yield	
		3-NH_2-β-CyD / mg (mmol)	Type of functionalization site	Functionalization site / mg (mmol)	DCC / mg (mmol)	HOBt•H_2O / mg (mmol)	Total amount of DMF/ mL	acetone / mL	/ mg (mmol)	/ %
BA-β-CyD	$C_{49}H_{75}NO_{35}$	226.8 (0.2)	benzoic acid	29.31 (0.24)	61.50 (0.30)	46.50 (0.30)	20 (10+10)	1000	171 (0.14)	69.1
2-HBA-β-CyD	$C_{49}H_{75}NO_{36}$	56.7 (0.05)	2-hydroxybenzoic acid	8.29 (0.06)	15.38 (0.08)	11.63 (0.08)	15 (10+5)	500	49 (0.04)	78.2
3-HBA-β-CyD	$C_{49}H_{75}NO_{36}$	56.7 (0.05)	3-hydroxybenzoic acid	8.29 (0.06)	15.38 (0.08)	11.63 (0.08)	10 (5+5)	500	43 (0.03)	68.6
4-HBA-β-CyD	$C_{49}H_{75}NO_{36}$	226.8 (0.2)	4-hydroxybenzoic acid	33.15 (0.24)	61.50 (0.30)	46.50 (0.30)	20 (10+10)	1000	261 (0.21)	105.0
2,5-DHBA-β-CyD	$C_{49}H_{75}NO_{37}$	113.4 (0.1)	2,5-dihydroxybenzoic acid	18.50 (0.12)	30.53 (0.15)	23.35 (0.15)	15 (10+5)	1500	103 (0.08)	40.5
2,6-DHBA-β-CyD	$C_{49}H_{75}NO_{37}$	56.7 (0.05)	2,6-dihydroxybenzoic acid	9.25 (0.06)	15.38 (0.08)	11.63 (0.08)	10 (5+5)	500	22 (0.02)	34.7
3,4-DHBA-β-CyD	$C_{49}H_{75}NO_{37}$	226.8 (0.20)	3,4-dihydroxybenzoic acid	36.99 (0.24)	61.50 (0.30)	46.50 (0.30)	20 (10+10)	1000	156 (0.12)	61.4
3,5-DHBA-β-CyD	$C_{49}H_{75}NO_{37}$	56.7 (0.05)	3,5-dihydroxybenzoic acid	9.25 (0.06)	15.38 (0.08)	11.63 (0.08)	10 (5+5)	500	49 (0.04)	77.2
3,4,5-THBA-β-CyD	$C_{49}H_{75}NO_{38}$	226.8 (0.2)	3,4,5-trihydroxybenzoic acid	40.83 (0.24)	61.50 (0.30)	46.50 (0.30)	20 (10+10)	1000	249 (0.19)	96.9
3,4-DHCA-β-CyD	$C_{51}H_{77}NO_{37}$	56.7 (0.05)	3,4-dihydroxycinnamic acid	10.79 (0.06)	15.38 (0.08)	11.63 (0.08)	10 (5+5)	500	47 (0.04)	72.6

2. CVs and DPVs of functionalized CyDs and Fc/functionalized CyDs

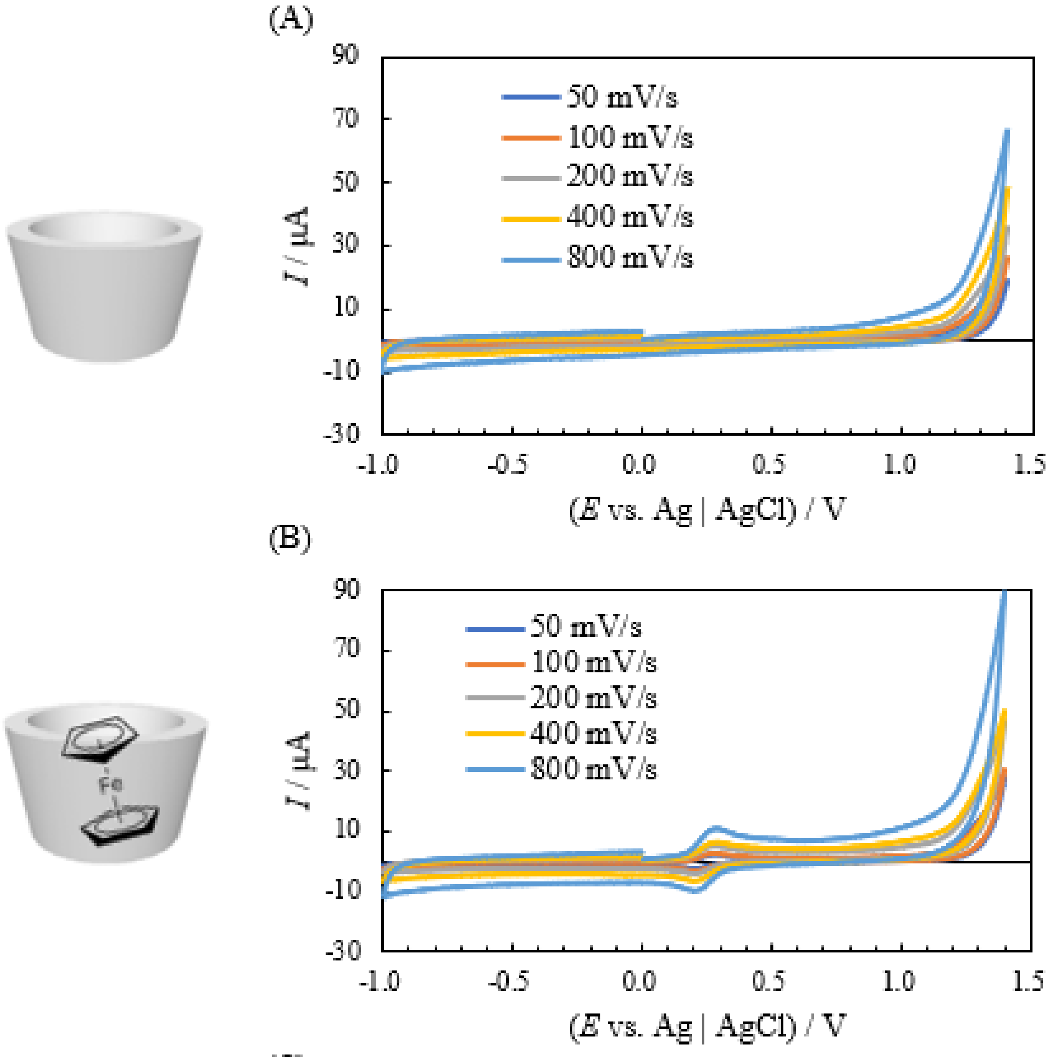

Figure. S20. CVs of functionalized CyD and its Fc inclusion complexes. (A) *β*-CyD, (B) Fc/*β*-CyD, (C) 2-HBA-*β*-CyD, (D) Fc/2-HBA-*β*-CyD, (E) 3-HBA-*β*-CyD, (F) Fc/3-HBA-*β*-CyD, (G) 2,5-DHBA-*β*-CyD, (H) Fc/2,5-DHBA-*β*-CyD, (I) 2,6-DHBA-*β*-CyD, (J) Fc/2,6-DHBA-*β*-CyD, (K) 3,4,5-THBA-*β*-CyD, (L) Fc/3,4,5-THBA-*β*-CyD, (M) 3,4-DHCA-*β*-CyD (N) Fc/3,4-DHCA-*β*-CyD. The measurements were performed in 90% H_2O/10% CH_3OH (v/v). [functionalized CyD] = 1.5 mM, [Fc] = 0.5 mM, [NaCl] = 0.1 M, [phosphate] = 50 mM, pH 8.6 at r.t. Scan rate = 0.05, 0.10, 0.20, 0.40, and 0.80 V s^{-1}. WE: sGCDE (ϕ=3.0 mm), RE: Ag|AgCl $_{\text{3M NaCl aq.}}$, CE: Pt coil.

(C)

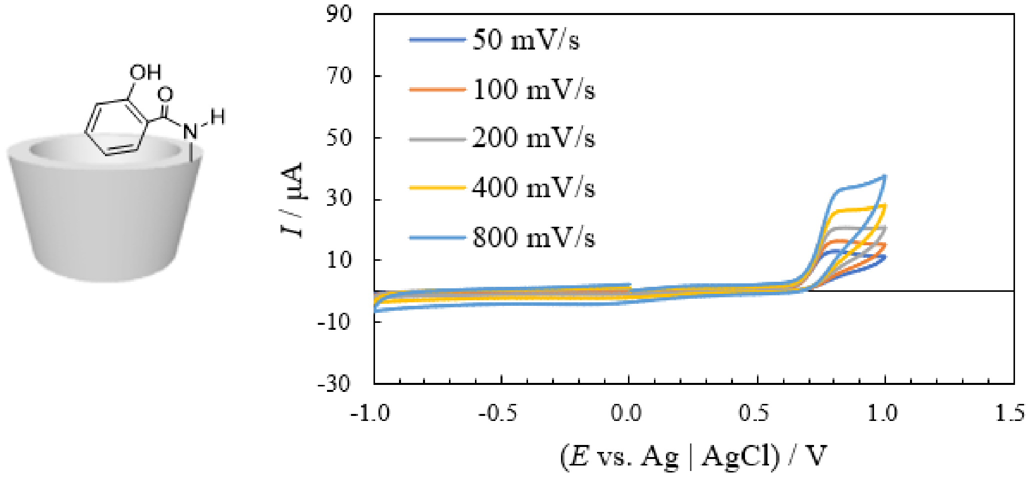

(D)

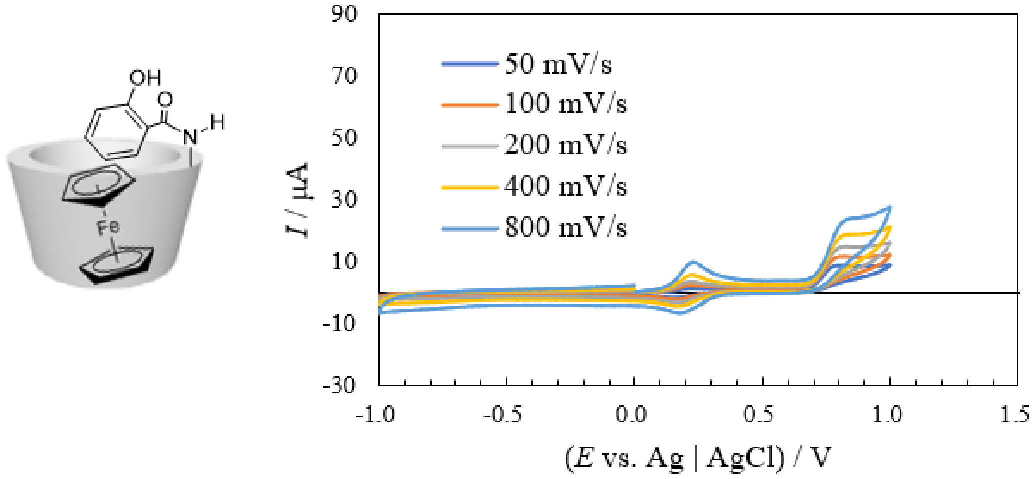

(E)

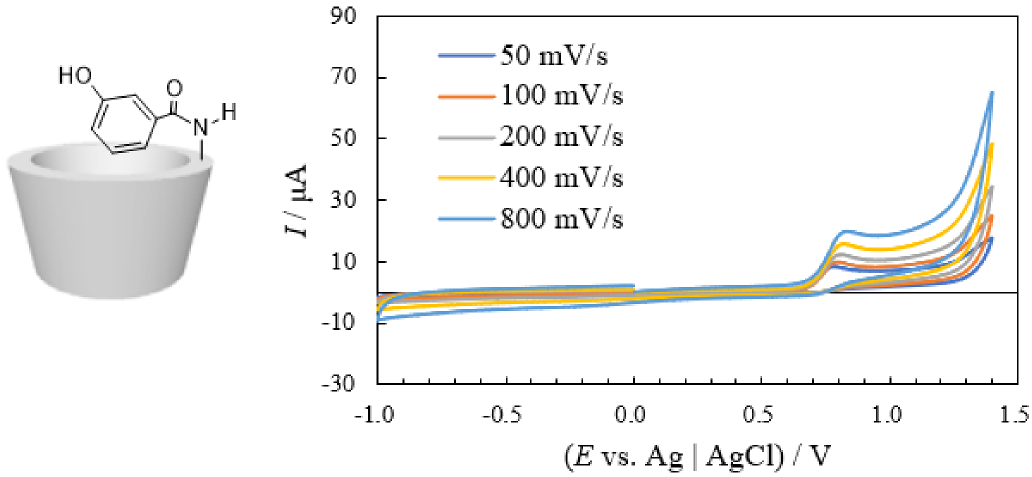

Figure. S20. CVs of functionalized CyD and its Fc inclusion complexes (continued).

(F)

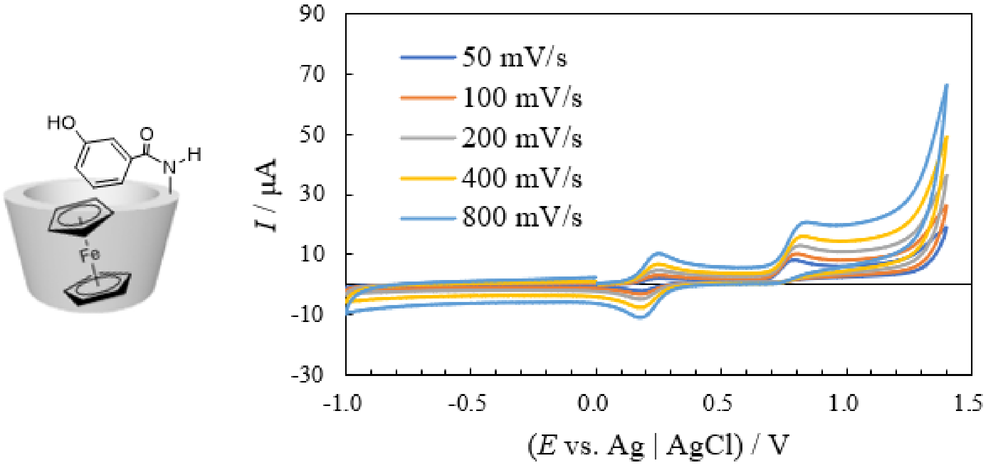

(G)

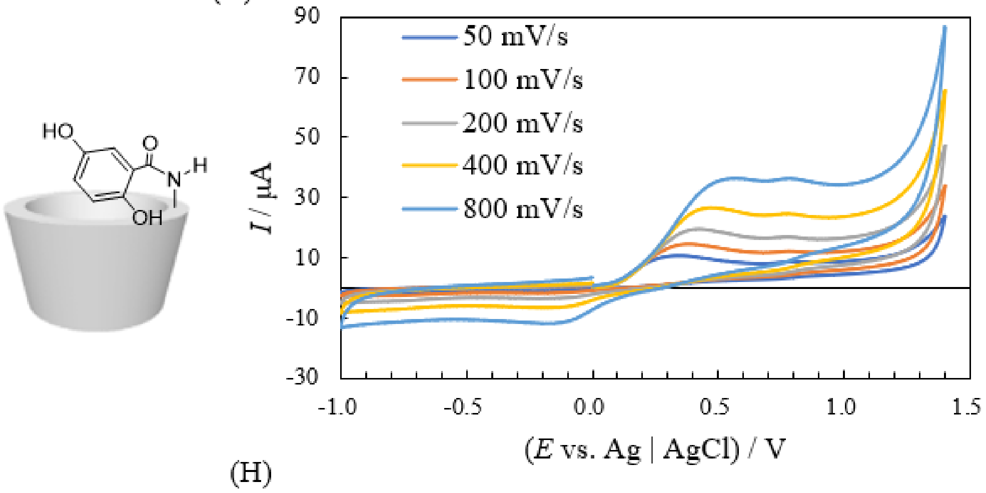

(H)

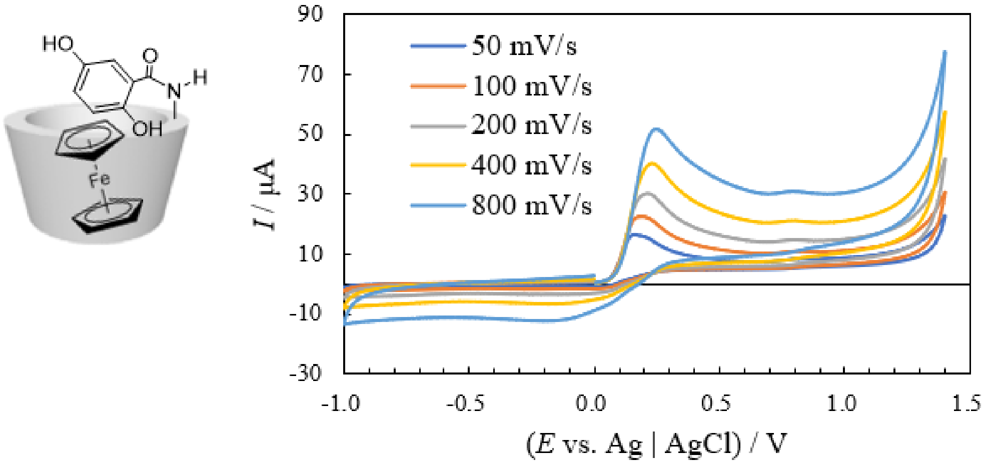

Figure. S20. CVs of functionalized CyD and its Fc inclusion complexes (continued).

(I)

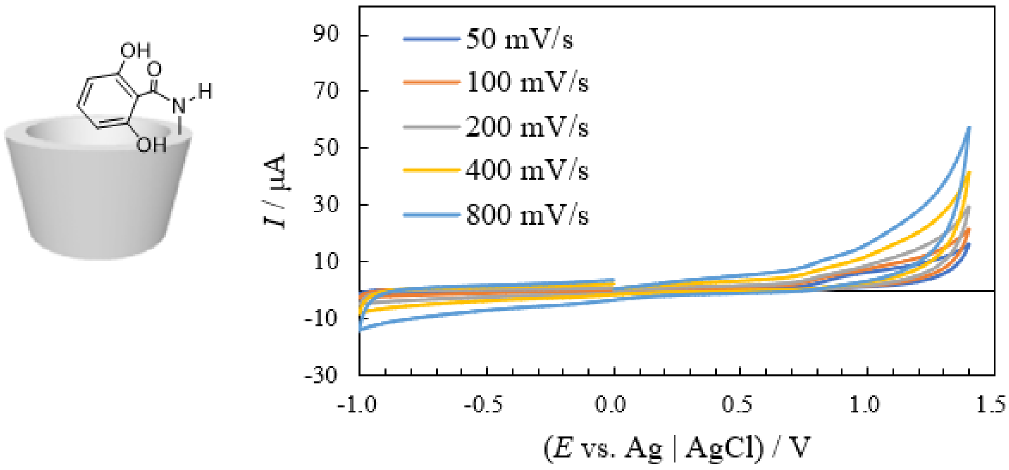

(J)

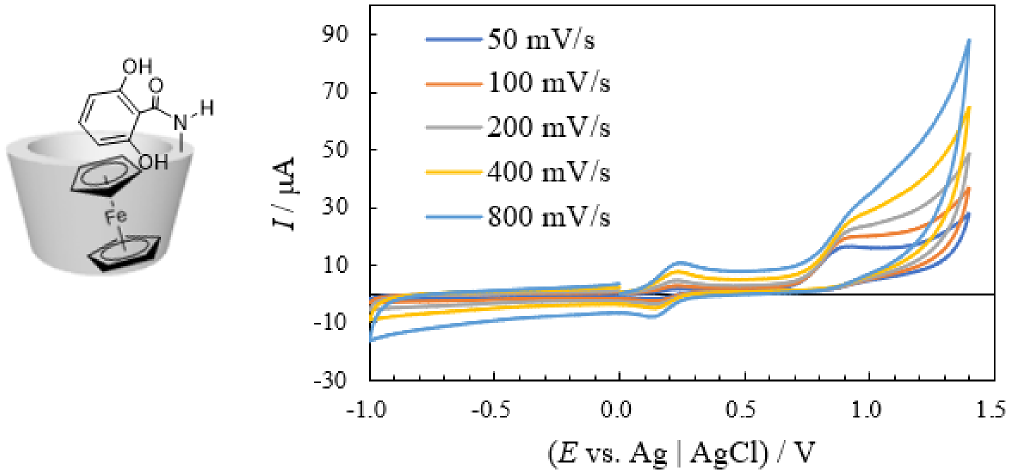

(K)

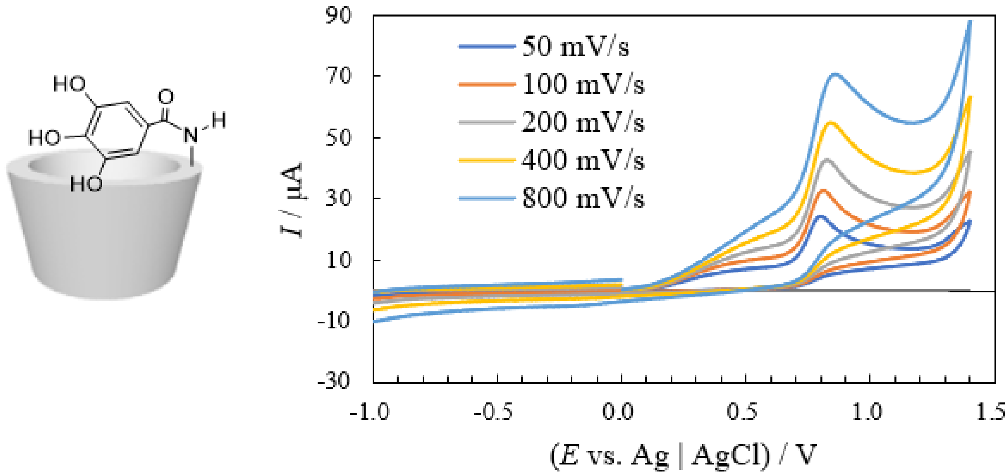

Figure. S20. CVs of functionalized CyD and its Fc inclusion complexes (continued).

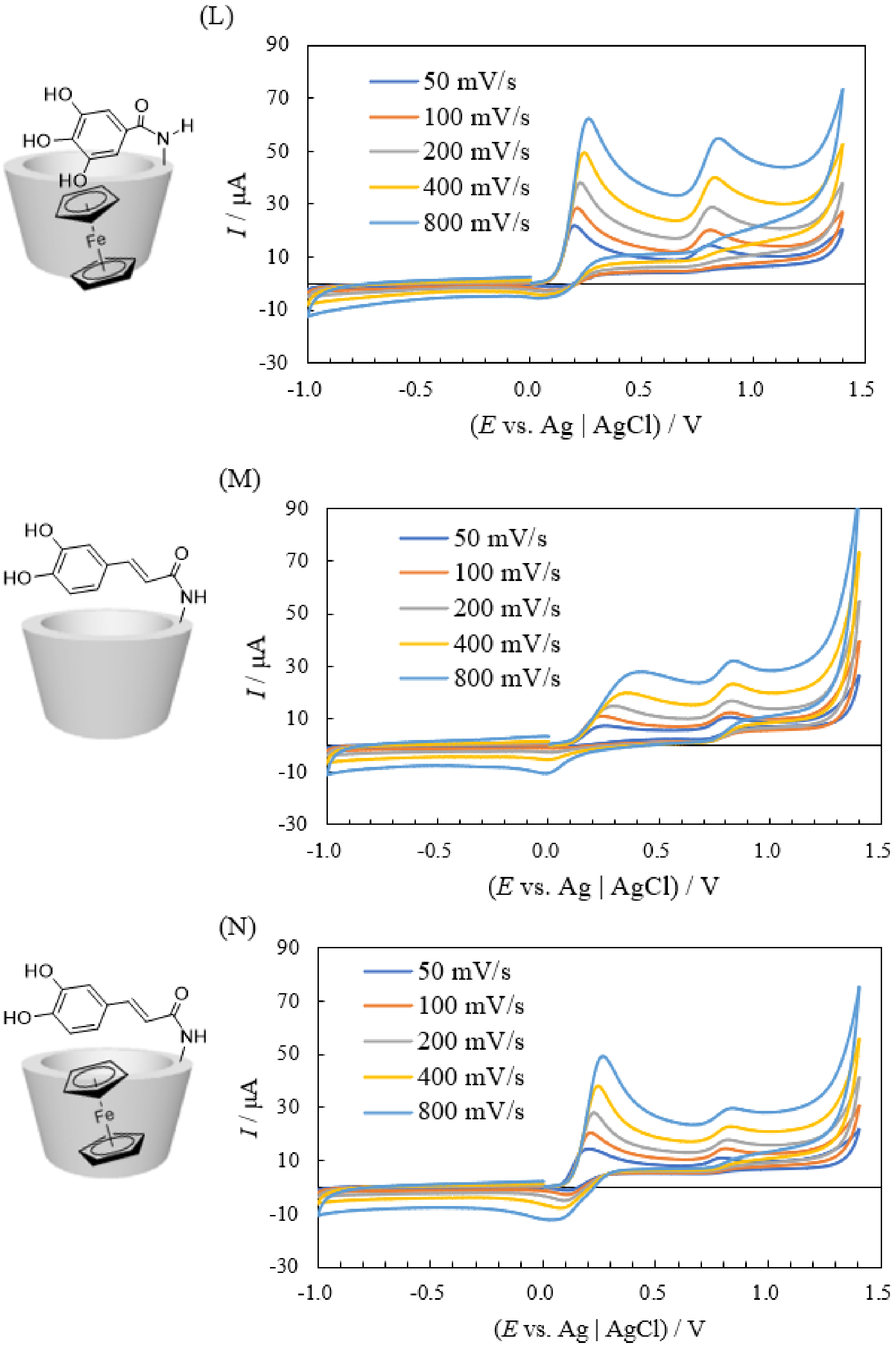

Figure. S20. CVs of functionalized CyD and its Fc inclusion complexes (continued).

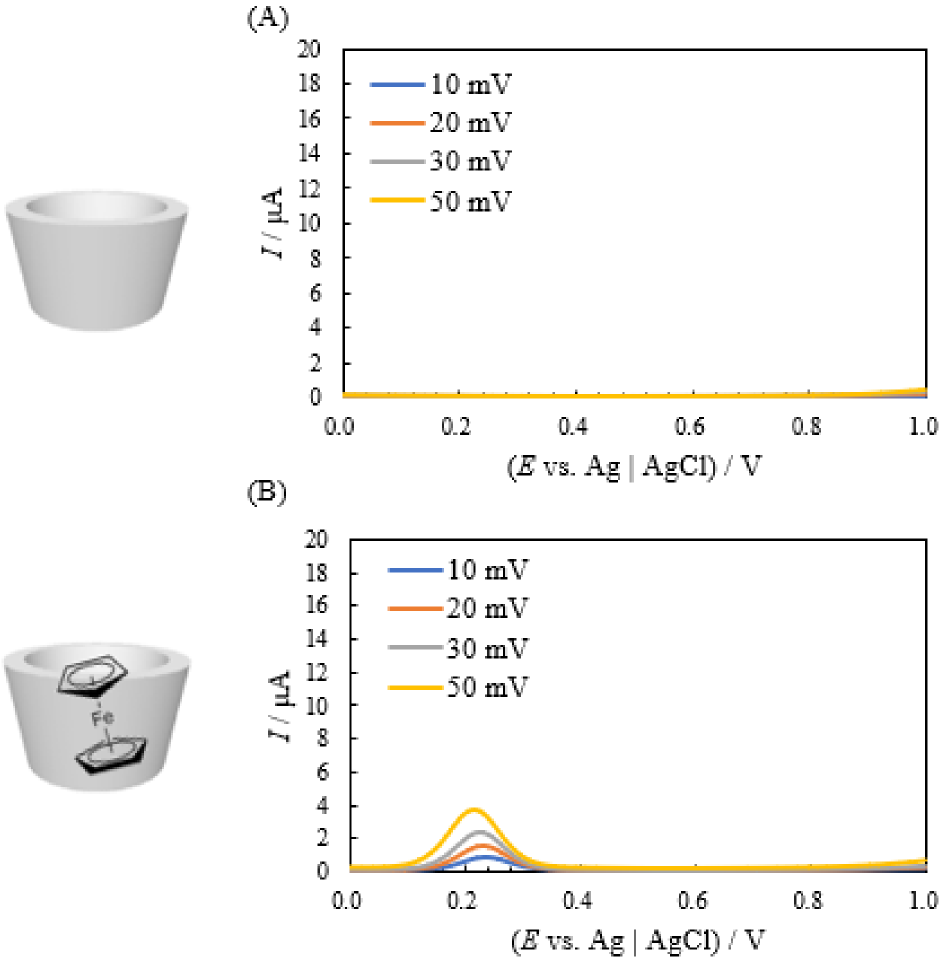

Figure. S21. DPVs of functionalized CyD and its Fc inclusion complexes. (A) β-CyD, (B) Fc/β-CyD, (C) 2-HBA-β-CyD, (D) Fc/2-HBA-β-CyD, (E) 3-HBA-β-CyD, (F) Fc/3-HBA-β-CyD, (G) 2,5-DHBA-β-CyD, (H) Fc/2,5-DHBA-β-CyD, (I) 2,6-DHBA-β-CyD, (J) Fc/2,6-DHBA-β-CyD, (K) 3,4,5-THBA-β-CyD, (L) Fc/3,4,5-THBA-β-CyD, (M) 3,4-DHCA-β-CyD (N) Fc/3,4-DHCA-β-CyD. The measurements were performed in 90% H_2O/10% CH_3OH (v/v). [functionalized CyD] = 1.5 mM, [Fc] = 0.5 mM, [NaCl] = 0.1 M, [phosphate] = 50 mM, pH 8.6 at r.t. Pulse amplitude = 10, 20, 30, and 50 mV, pulse step = 5 mV, a modulation time (pulse width) = 60 ms, interval time (pulse period) of 200 ms, and quiet time = 5 s. WE: sGCDE (ϕ=3.0 mm), RE: Ag|AgCl $_{3M\ NaCl\ aq.}$, CE: Pt coil.

(C)

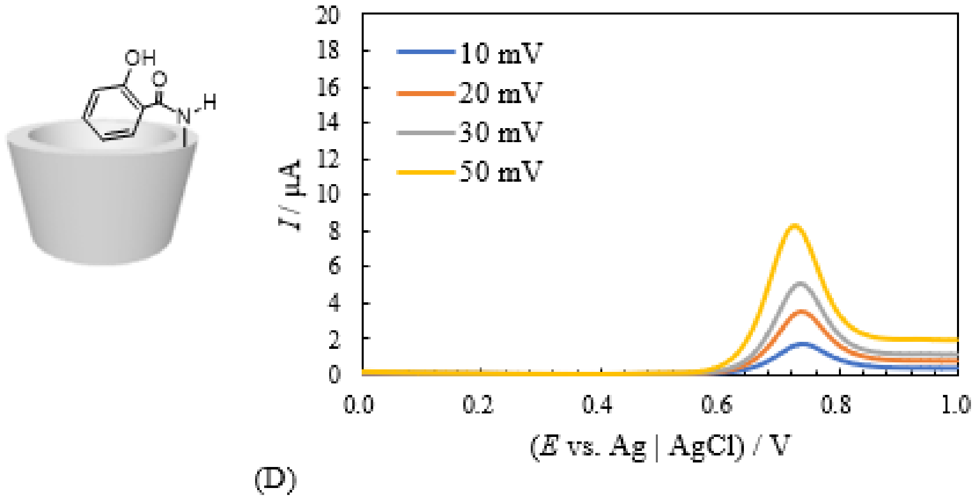

(D)

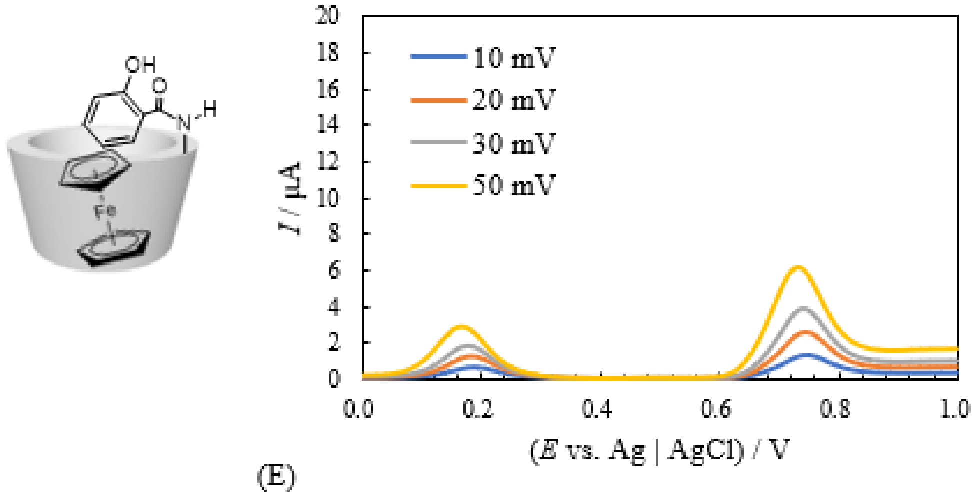

(E)

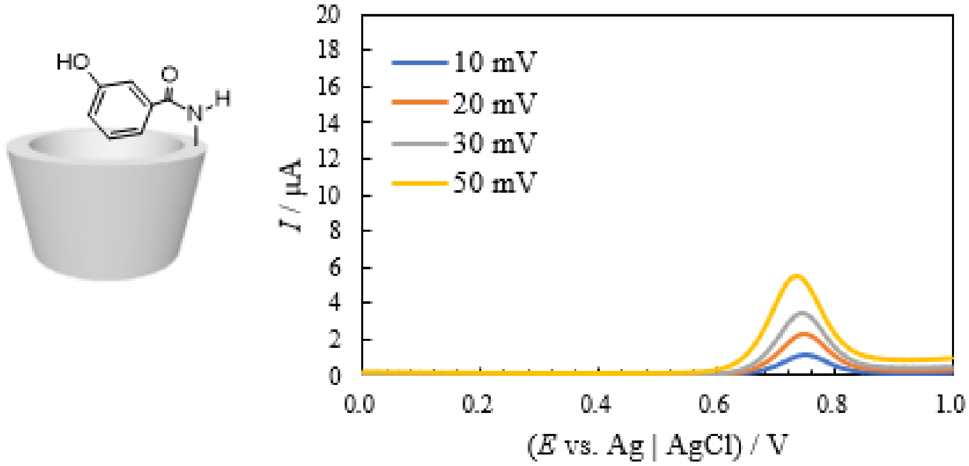

Figure. S21. DPVs of functionalized CyD and its Fc inclusion complexes (continued).

(F)

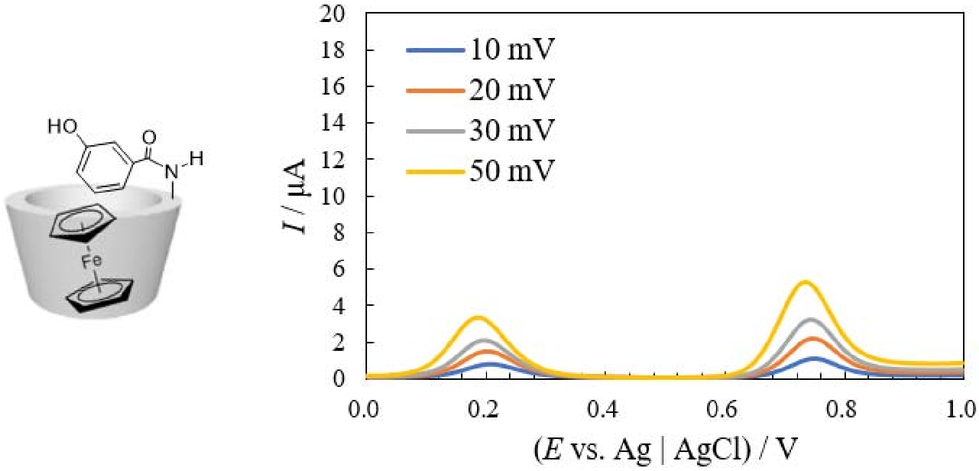

(G)

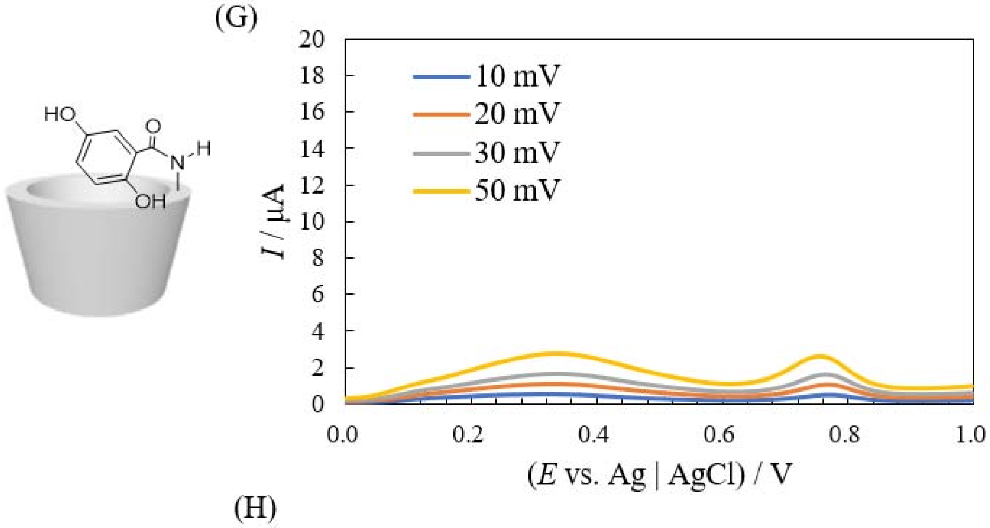

(H)

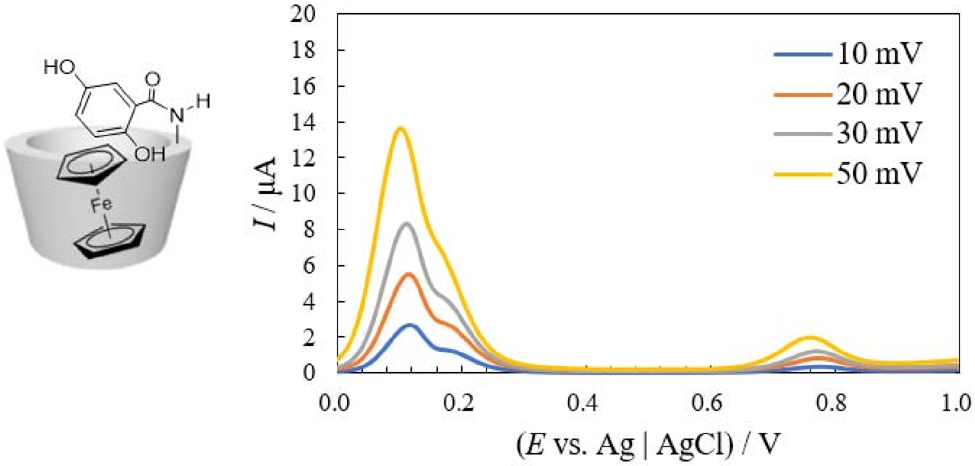

Figure. S21. DPVs of functionalized CyD and its Fc inclusion complexes (continued).

(I)

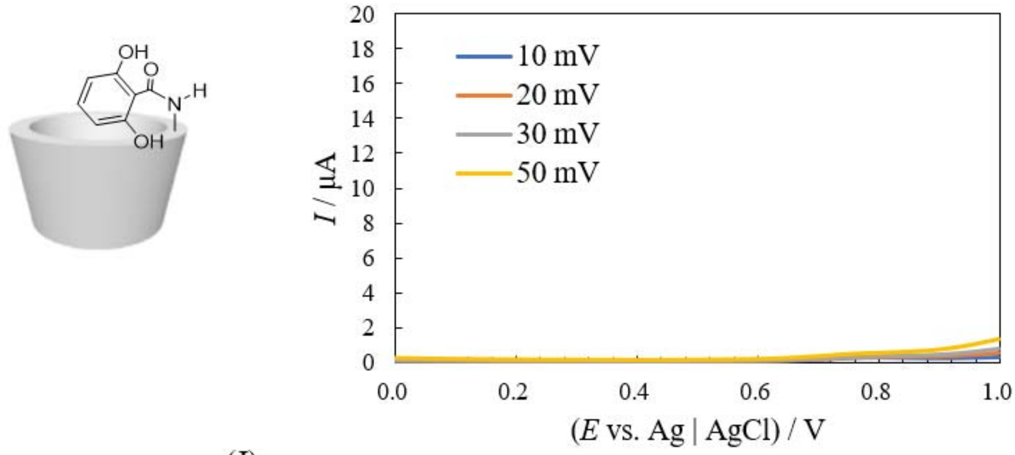

(J)

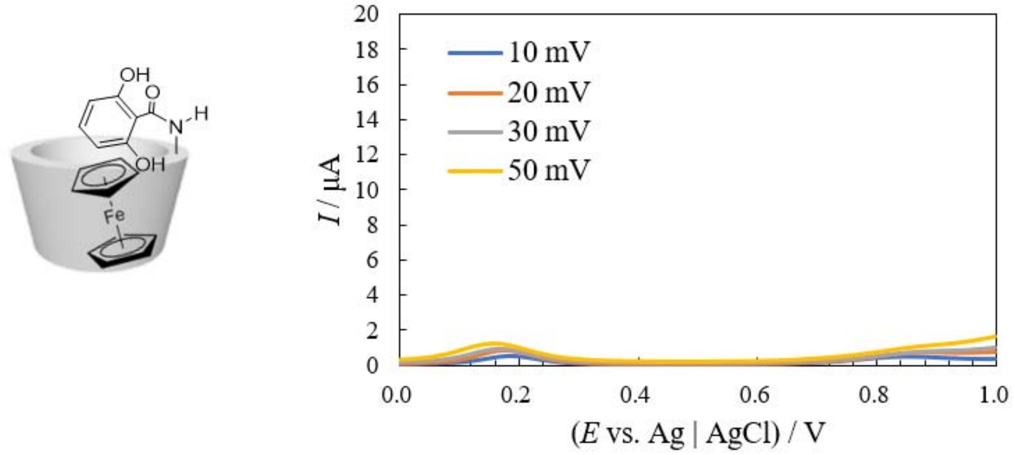

(K)

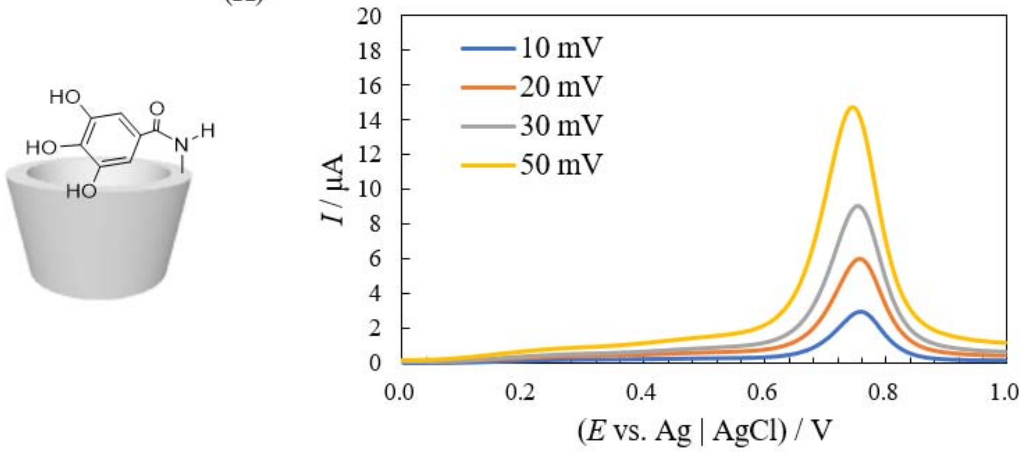

Figure. S21. DPVs of functionalized CyD and its Fc inclusion complexes (continued).

[illegible]

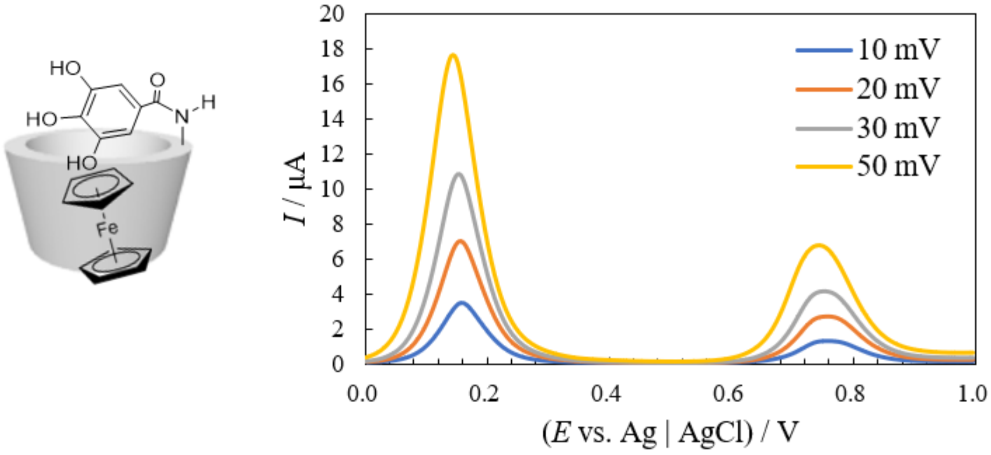

(M)

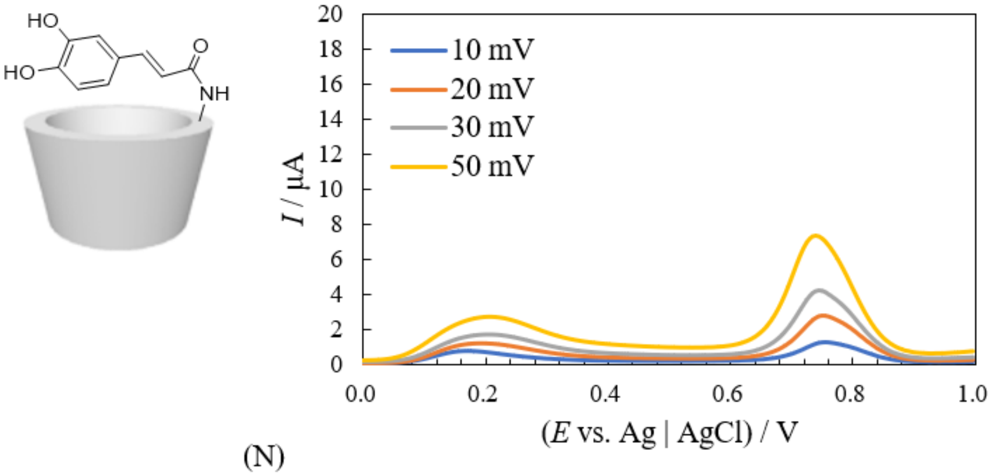

(N)

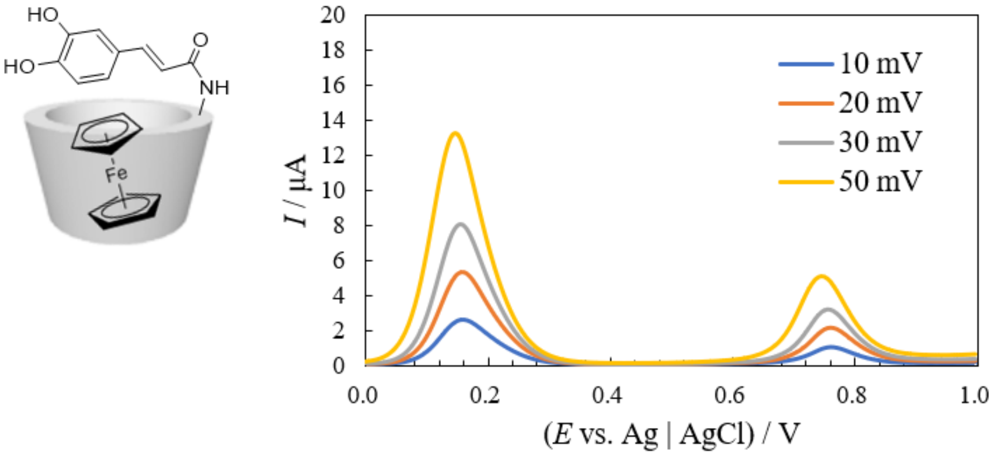

Figure. S21. DPVs of functionalized CyD and its Fc inclusion complexes (continued).

3. Confirmation of inclusion complex formation

In the ^{1}H NMR spectrum, a signal originating from the hydrogen of the cyclopentadienyl ring of Fc (*, Figure. 22) was observed at 4.015 ppm in the presence of Fc alone ([48], Figure. S2(A)). Signals of Fc in the presence of 2-HBA-β-CyD, 3-HBA-β-CyD, 2,5-DHBA-β-CyD, 2,6-DHBA-β-CyD, 3,4,5-THBA-β-CyD, and 3,4-DHCA-β-CyD were observed at 3.940, 3.797, 3.940, 4.098, 3.878, and 3.871 ppm, respectively. The observed shift of the Fc signal in the presence of functionalized CyD indicates the formation of inclusion complexes.

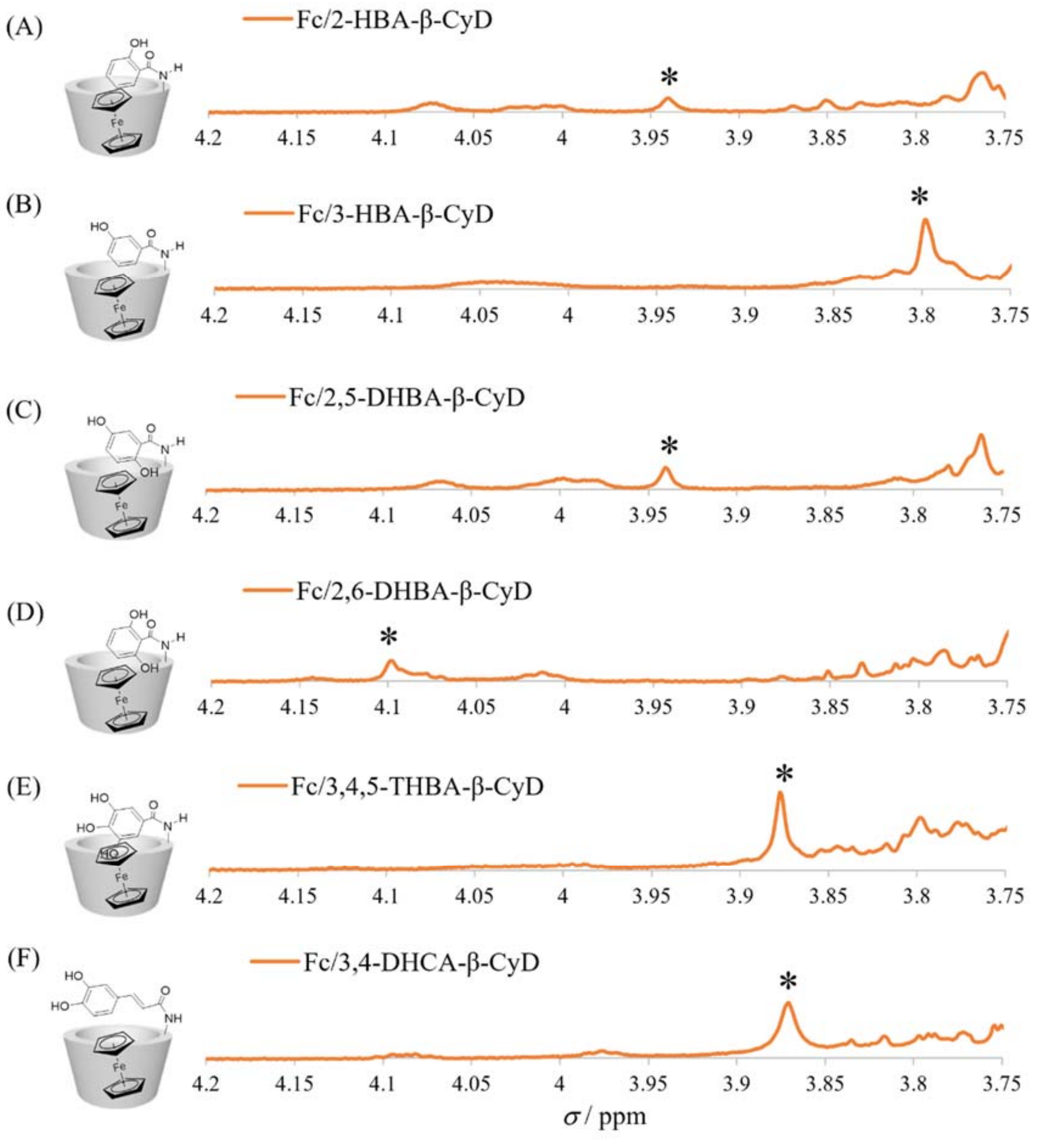

Figure. S22. ^{1}H NMR spectrum of Fc and Fc/functionalized β-CyD, [Fc] = 0.5 mM, [functionalized β-CyD] = 1.5 mM in 90% D_2O/10% CD_3OH (v/v) at 298 K, 500 MHz, 16 scans.

4. The pH dependence of the response to boron

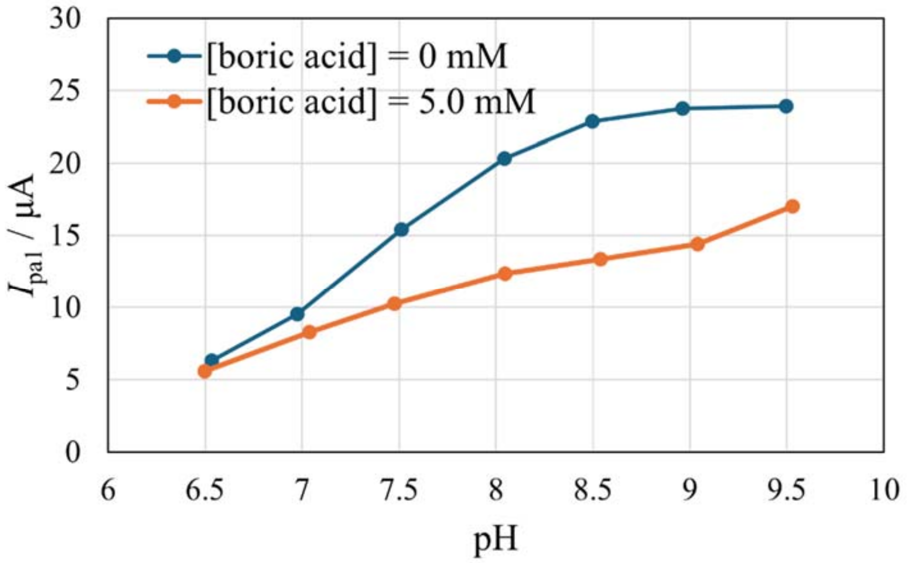

Figure. S23. Plots of I_{pa1} as functions of pH for solutions with and without boron.

5. Effect of adding boric acid aqueous solution on pH

The relationship between boron concentration and pH after adding boric acid aqueous solution is shown in Figure. S24. The pH value remained unchanged after adding boric acid aqueous solution.

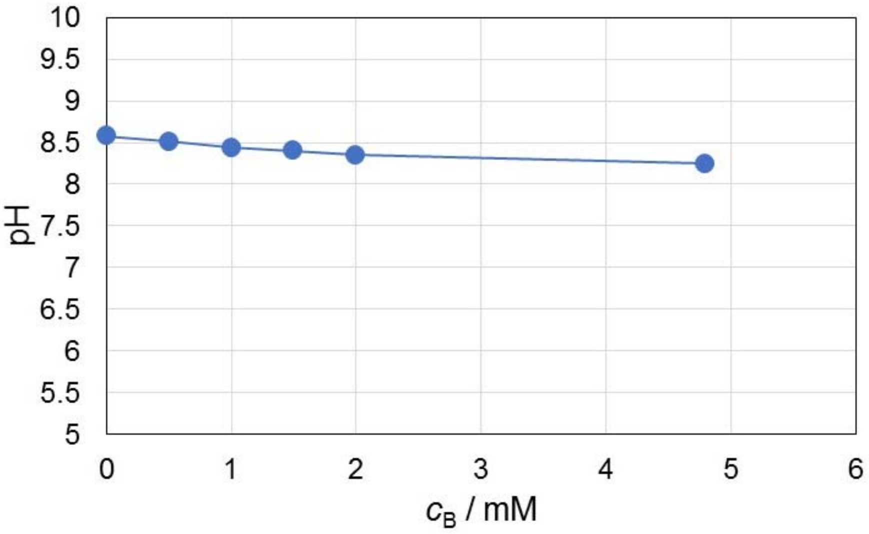

Figure. S24. Plot of pH against boron concentration.

6. CV stability of Fc/3,4-DHBA-*β*-CyD

I_{pa1} was not decreased when 12 μL of ultrapure water was added (Figure. S25). We have reported that CVs were not altered after ten consecutive CV measurements [48]. From these results, the peak current decrease when boric acid aqueous solution was added was caused by the interaction between boric acid and the inclusion complex.

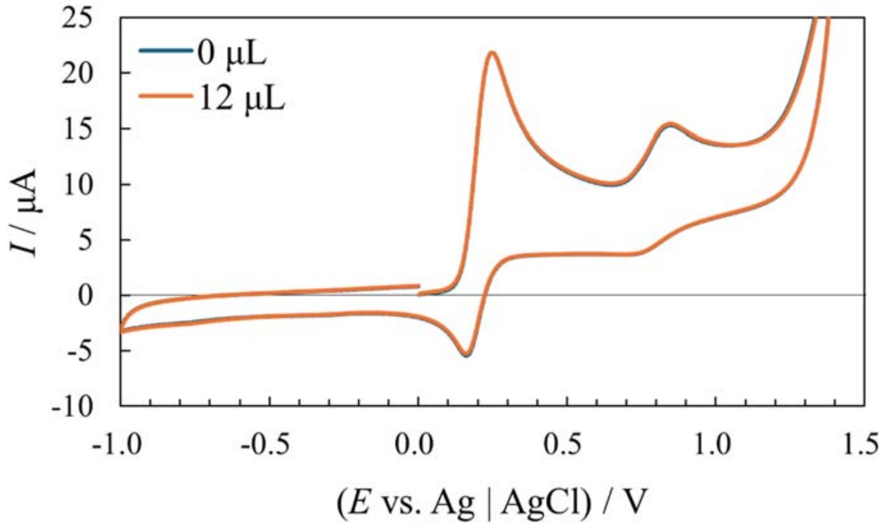

Figure. S25. CV of Fc/3,4-DHBA-*β*-CyD in 10% CH_3OH/90% H_2O (v/v) with the addition of 12 μL of water. [Fc] = 0.5 mM, [3,4-DHBA-*β*-CyD] = 1.5 mM, [NaCl] = 0.1 M, [phosphate] = 50 mM. Scan rate = 0.1 V s^{-1}, pH 8.6 at r.t. WE: sGCDE (ϕ=3.0 mm), RE: Ag|AgCl $_{3M\ NaCl\ aq.}$, CE: Pt coil.

7. Spike test recovery with puddle water, river water, and tap water

Spike recovery tests were conducted using puddle water, river water, and tap water samples. These real samples were suction-filtered using a membrane filter to remove particles and other contaminants before being used in the measurements. As shown in Figures. S26 and S27, the same-level response was observed for the real samples as ultrapure water, and the linearity of the calibration curve and the recovery rate were good, indicating that boron in these real samples can be determined using Fc/3,4-DHBA-*β*-CyD.

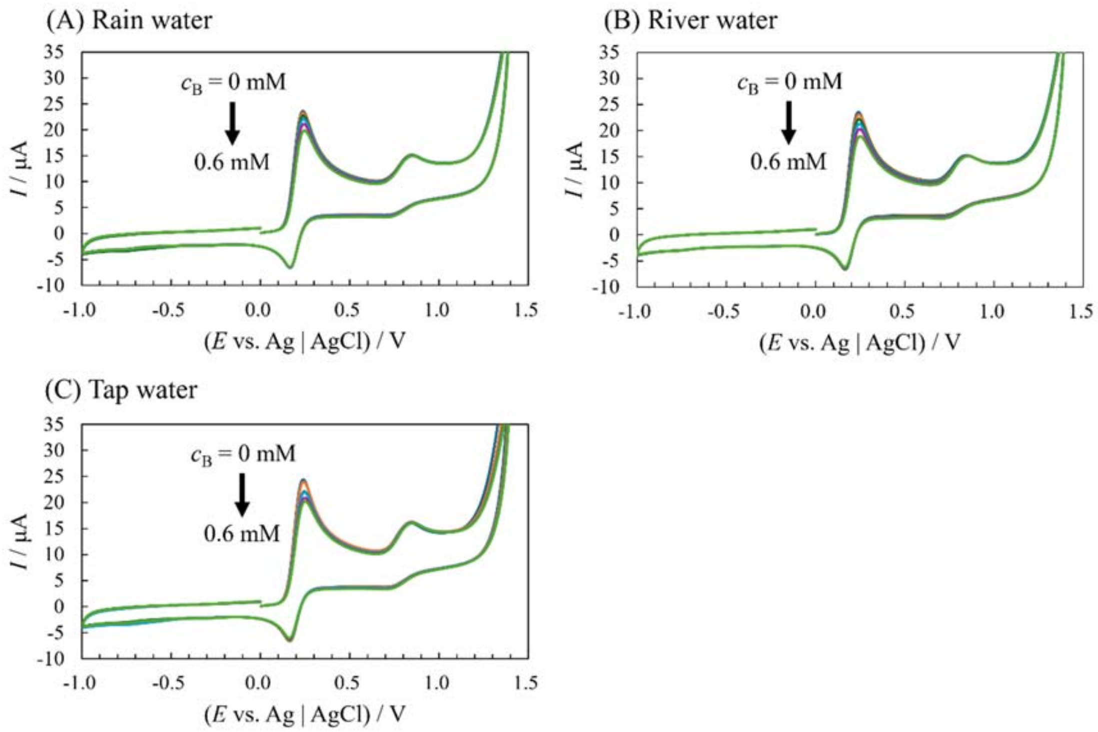

Figure. S26. CVs of Fc/3,4-DHBA-*β*-CyD in 90% (A) puddle water, (B) river water, or (C) tap water/10% CH_3OH (v/v) in various c_B (0 – 0.6 mM) at pH 8.6, r.t. [Fc] = 0.5 mM, [3,4-DHBA-*β*-CyD] = 1.5 mM, [NaCl] = 0.1 M, [phosphate] = 50 mM Scan rate = 0.1 V s^{-1}. WE: sGCDE (ϕ=3.0 mm), RE: Ag|AgCl $_{3M\ NaCl\ aq.}$, CE: Pt coil.

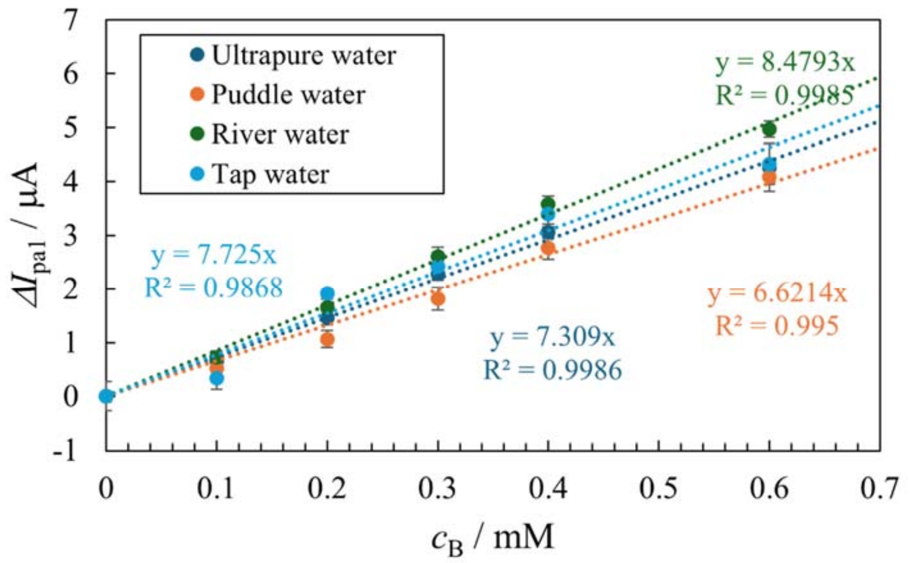

Figure. S27. The result of boron determination using Fc/3,4-DHBA-β-CyD in real samples. Plot of ΔI_{pa1} against c_{B} (n = 3). $\Delta I_{\mathrm{pa1}} = (I_{\mathrm{pa1\ without\ boric\ acid}}) - (I_{\mathrm{pa1\ with\ boric\ acid}})$.

8. Effects of coexisting ions

8-1. Effects of coexisting ions on CV of Fc/3,4-DHBA-β-CyD

The effects of coexisting ions on the CV of Fc/3,4-DHBA-β-CyD were investigated. All coexisting ions were added at a concentration of 5.0 mM. The results are shown in Figure. S28. The blue line in the graph shows the CVs in the absence of coexisting ions. The red line shows the CVs in the presence of coexisting ions, Al^{3+}, Ba^{2+}, Ca^{2+}, Cd^{2+}, Cu^{2+}, K^{+}, Li^{+}, Mg^{2+}, Ni^{2+}, CH_3COO^{-}, ClO_4^{-}, F^{-}, I^{-}, NO_3^{-}, and SO_4^{2-}. I_{pa1} was little affected by the presence of Ba^{2+}, Ca^{2+}, Cd^{2+}, K^{+}, Li^{+}, Mg^{2+}, CH_3COO^{-}, ClO_4^{-}, F^{-}, NO_3^{-}, and SO_4^{2-}. On the other hand, the CVs changed significantly in the presence of Al^{3+}, Cu^{2+}, Ni^{2+}, and I^{-}.

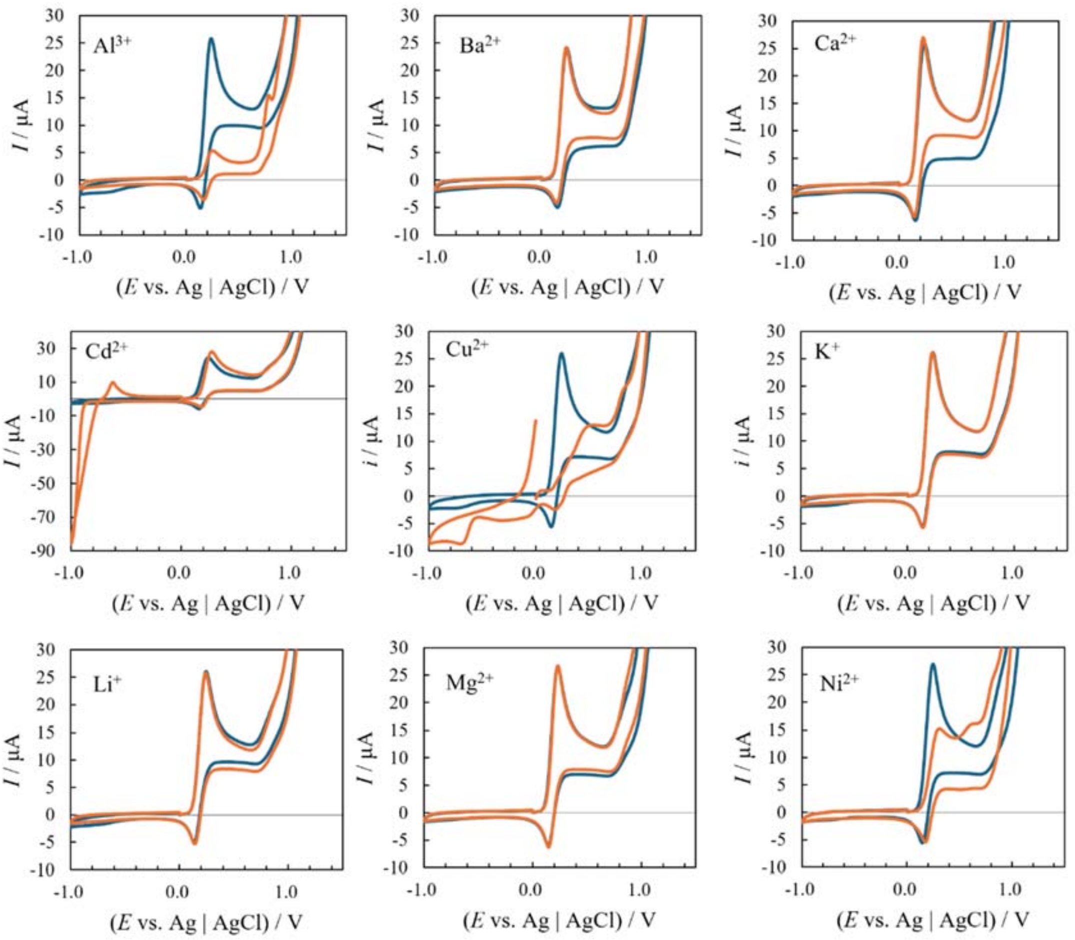

Figure. S28. CVs of Fc/3,4-DHBA-β-CyD in 90% H_2O/10% CH_3OH (v/v) at pH 8.6, r.t. [Fc] = 0.5 mM, [3,4-DHBA-β-CyD] = 1.5 mM, [NaCl] = 0.1 M, [HEPES] = 50 mM, [coexisting ion] = 0 mM (blue line) or 5.0 mM (red line). Scan rate = 0.1 V s^{-1}. WE: sGCDE (ϕ=3.0 mm), RE: Ag|AgCl $_{3M\ NaCl\ aq.}$, CE: Pt coil.

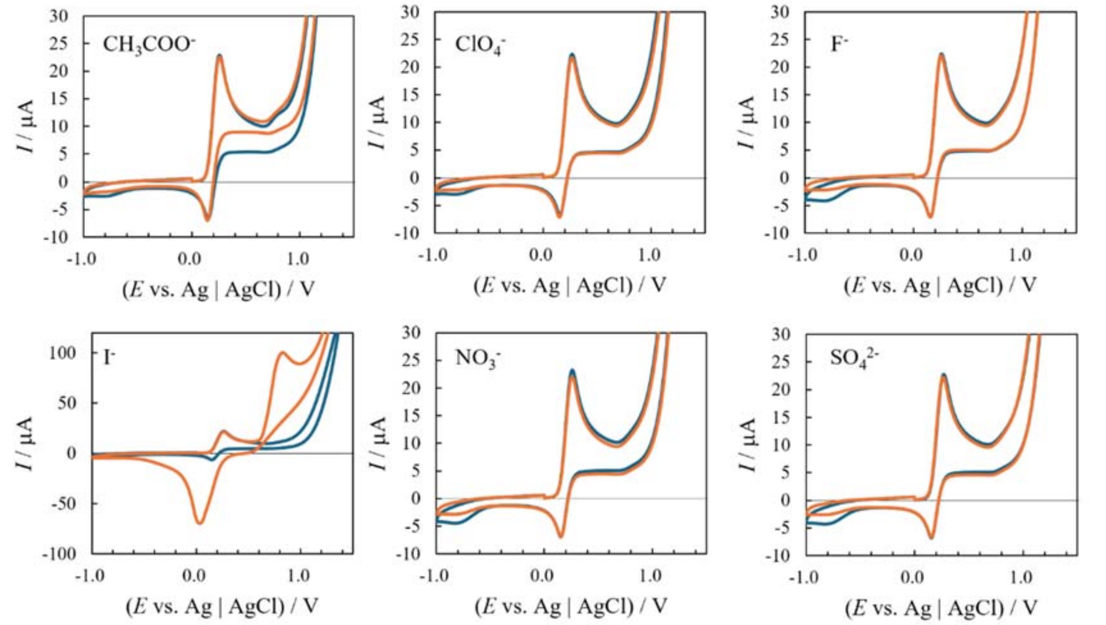

Figure. S28. CVs of Fc/3,4-DHBA-β-CyD in 90% H_2O/10% CH_3OH (v/v) at pH 8.6, r.t. (continued).

As shown in Figure. S29, selectivity for boron was investigated using the results of Figure. S28. Fc/3,4-DHBA-β-CyD did not respond to Ba^{2+}, Ca^{2+}, Cd^{2+}, K^{+}, Li^{+}, Mg^{2+}, CH_3COO^{-}, ClO_4^{-}, F^{-}, I^{-}, NO_3^{-}, or SO_4^{2-}. However, it responded to Al^{3+}, Cu^{2+}, and Ni^{2+}.

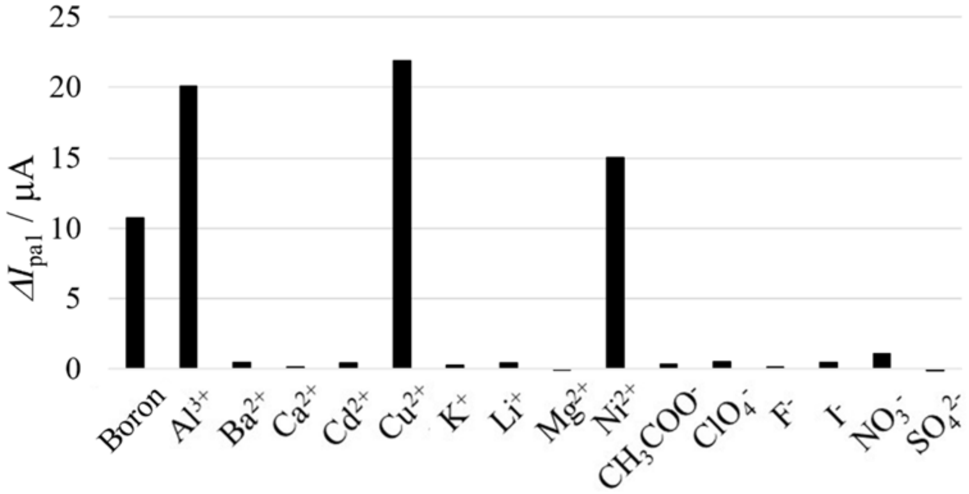

Figure. S29. Comparison of response to boron or other ions. CVs were performed in 90% H_2O/10% CH_3OH (v/v) at pH 8.6, r.t. [Fc] = 0.5 mM, [3,4-DHBA-β-CyD] = 1.5 mM, [NaCl] = 0.1 M, [phosphate] = 50 mM, [boron or other ions] = 0 mM or 5.0 mM. $\Delta I_{pa1} = (I_{pa1\ \text{without boric acid or other ions}}) - (I_{pa1\ \text{with 5.0 mM of boric acid or other ions}})$.

8-2. Effects of coexisting ions on the reactivity of Fc/3,4-DHBA-*β*-CyD to boric acid

The effect of coexisting ions on the response to boron was examined. Coexisting ions and boron were added at 5.0 mM. The red line shows the results in the absence of boron. The green line shows the results in the presence of boron. As shown in Figure. S30, the response to boron did not change in the presence of Ba^{2+}, Ca^{2+}, K^{+}, Li^{+}, Mg^{2+}, CH_3COO^{-}, ClO_4^{-}, F^{-}, NO_3^{-}, or SO_4^{2-}, meaning that the reactivity of Fc/3,4-DHBA-*β*-CyD to boron was not affected by these ions.

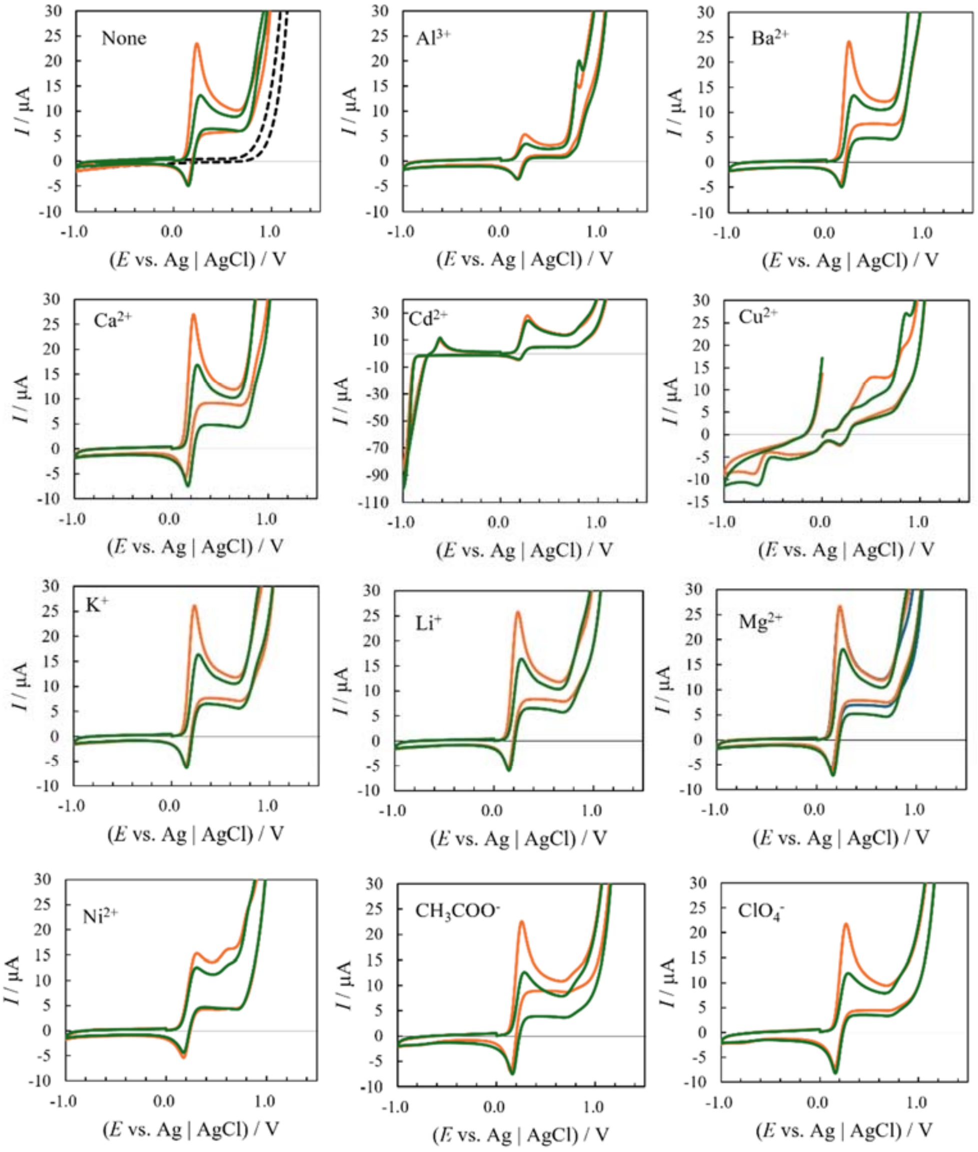

Figure. S30. CVs of Fc/3,4-DHBA-*β*-CyD in 90% H_2O/10% CH_3OH (v/v) at pH 8.6, r.t. [Fc] = 0.5 mM, [3,4-DHBA-*β*-CyD] = 1.5 mM, [NaCl] = 0.1 M, [HEPES] = 50 mM, [coexisting cation] = 5.0 mM, c_B = 0 mM (red line) or 5.0 mM (green line), scan rate = 0.1 V s^{-1}. WE: sGCDE (ϕ=3.0 mm), RE: Ag|AgCl $_{3M\ NaCl\ aq.}$, CE: Pt coil.

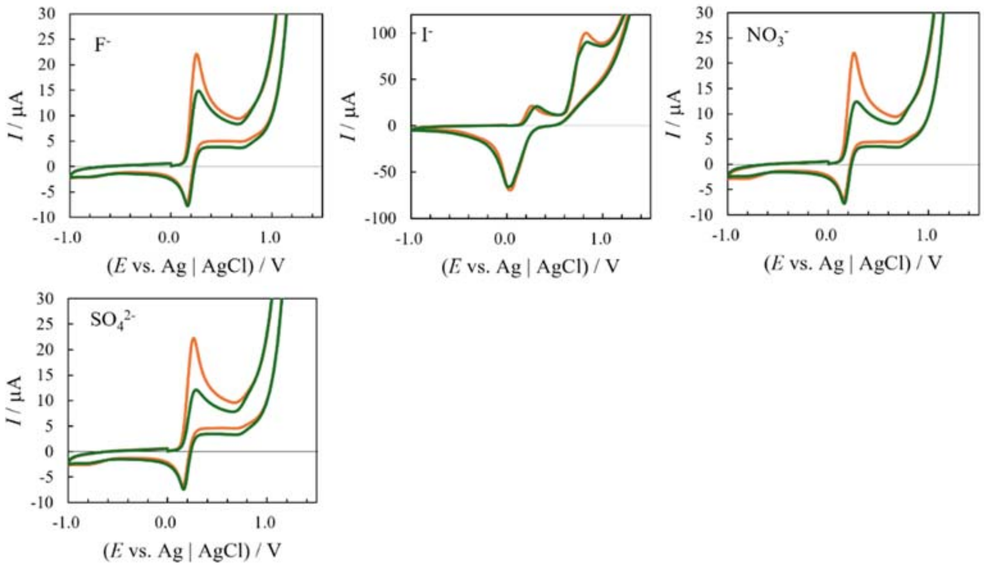

Figure. S30. CVs of Fc/3,4-DHBA-β-CyD in 90% H_2O/10% CH_3OH (v/v) at pH 8.6, r.t. (continued).

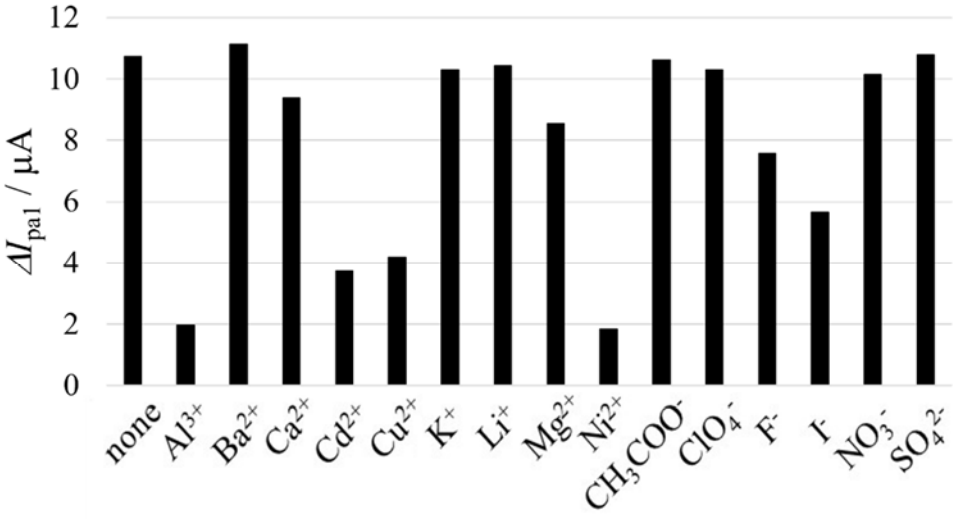

Figure. S31. Comparison of response to boric acid in the presence of coexisting ions. CVs were performed in 90% H_2O/10% CH_3OH (v/v) at pH 8.6 r.t. [Fc] = 0.5 mM, [3,4-DHBA-β-CyD] = 1.5 mM, [NaCl] = 0.1 M, [phosphate] = 50 mM, [coexisting ion] = 5.0 mM, c_B = 0 or 5.0 mM. $\Delta I_{pa1} = (I_{pa1\ \text{without boric acid}}) - (I_{pa1\ \text{with 5.0 mM of boric acid}})$.

9. Stability of reagents

The stability of reagents was investigated. The Fc-MeOH solution was prepared and stored in room temperature, and 3,4-DHBA-β-CyD was dissolved into 50 mM phosphate buffer-0.1 M NaCl aqueous solution degassed with argon gas and stored in an amber container at 3 °C in a refrigerator (Figure. S32). CVs were performed with or without 4.8 mM of boron 0, 21, 42, and 112 days after preparing the solution (Figure. S33). The response of Fc/3,4-DHBA-β-CyD to boron remained unchanged after 112 days.

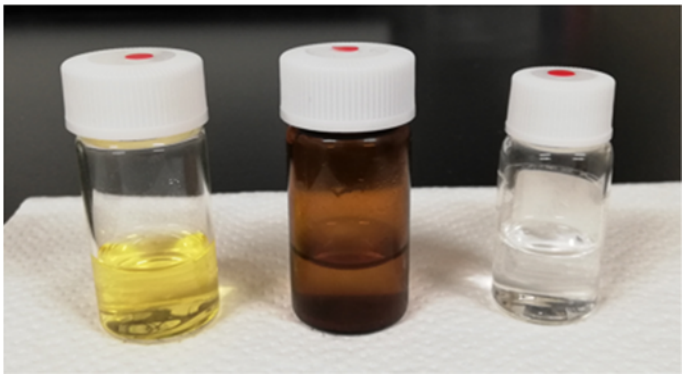

Figure. S32. Storage status of Fc-MeOH solution (left), 3,4-DHBA-β-CyD solution (center), and NaOH aqueous solution for adjusting pH (right). Fc-MeOH solution and NaOH aqueous solution were stored at r.t.

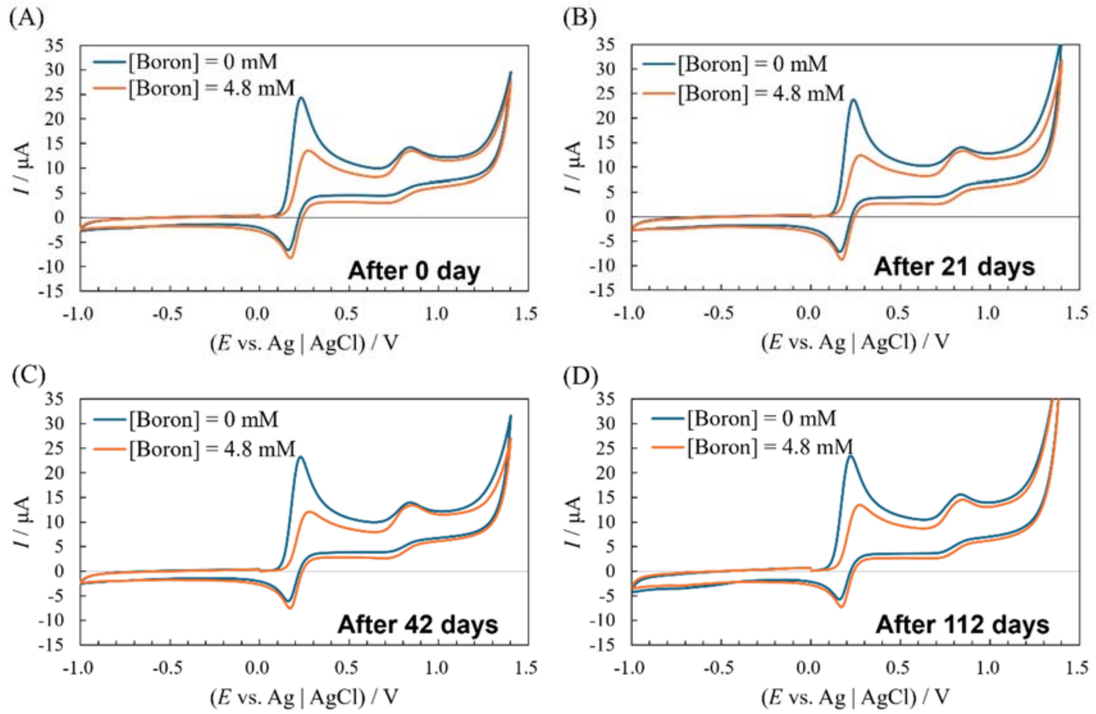

Figure. S33. CVs of Fc/3,4-DHBA-*β*-CyD aqueous solution (A) 0, (B) 21, (C) 42, and (D) 112 days after preparation, c_B = 0 mM (blue line) or 5 mM (red line) at pH 8.6, at r.t. Scan rate = 0.1 V s^{-1}. WE: sGCDE (ϕ=3.0 mm), RE: Ag|AgCl $_{3M\ NaCl\ aq.}$, CE: Pt coil.

10. The comparison with the previously reported boron determination methods

Table S2. The comparison with previously reported methods

Probe	Technique	LOD / mg B L^{-1}	Reference
Curucumin	Absorbance measurement (paper-based analytical devices)	0.2-0.8	[31]
Azomethine H	Absorbance measurement	0.02	[34]
	Square Wave Voltammetry	0.10	[35]
F-SA	Square Wave Voltammetry	0.03	[36]
Tiron	Differential Pulse Voltammetry	0.1	[37]
$[Ru^{II}(bpy)_2(dhphen)]^{2+}$	Spectrofluorometry	0.2	[38]
$[Ru^{III}(acac)_2(H_2thap)]$	Differential Pulse Voltammetry	1.03	[39]
Fc/3,4-DHBA-β-CyD	Cyclic Voltammetry	0.16	This work

11. Binding constant for the interaction between Fc/3,4-DHBA-*β*-CyD and boric acid

The absorption spectra of Fc/3,4-DHBA-*β*-CyD were measured at various boron concentrations (Figure. S34A), and the binding constants for interaction between boric acid and the inclusion complex were calculated by analysis of the Excel 2021 program (Figure. S34B). The absorbance around 250 nm decreased and the absorbance around 270 nm increased with increasing of boron concentration. The binding constant calculated from the absorbance decrease at 250 nm was approximately 1500 M^{-1}.

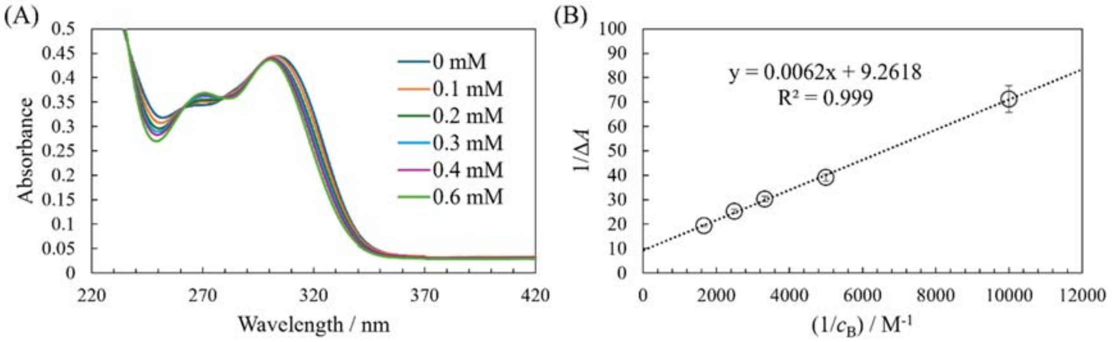

Figure. S34. (A) UV-vis spectrum of Fc/3,4-DHBA-*β*-CyD in 90% H_2O/10% CH_3OH (v/v) with various c_B (0 – 0.6 mM) at pH 8.6 at 25 °C. [3,4-DHBA-*β*-CyD] = 60 μM, [Fc] = 20 μM, [phosphate] = 50 mM. (b) Plots of $1/\Delta A$ as functions of $1/c_B$ (n = 3). ΔA = (Absorbance at 250 nm without boron) – (Absorbance at 250 nm with boron).

The binding constant was calculated from the absorbance at 250 nm against the concentration of boron (Figure. S34). Data were analyzed using the MS-Excel 2021 program based on the Benesi–Hildebrand method. The binding constant was calculated as approximately 1500 M^{-1} by plotting $1/\Delta A$ as a function of $1/c_B$ (Benesi-Hildebrand plot, Figure. S34B) and dividing the y-intercept of the approximate line by the slope value.

12. DPV response to boron

As shown in Figure. S35 and S36, DPVs of Fc/3,4-DHBA-β-CyD were conducted at various boron concentrations and pulse amplitudes (Figure. S35, S36, and Table S3). I_{pa1} was decreased with increasing c_B. LODs were calculated as 0.71, 0.90, and 1.87 mg B L^{-1} (Table S4), higher than the LOD 0.16 mg B L^{-1} calculated from the CV.

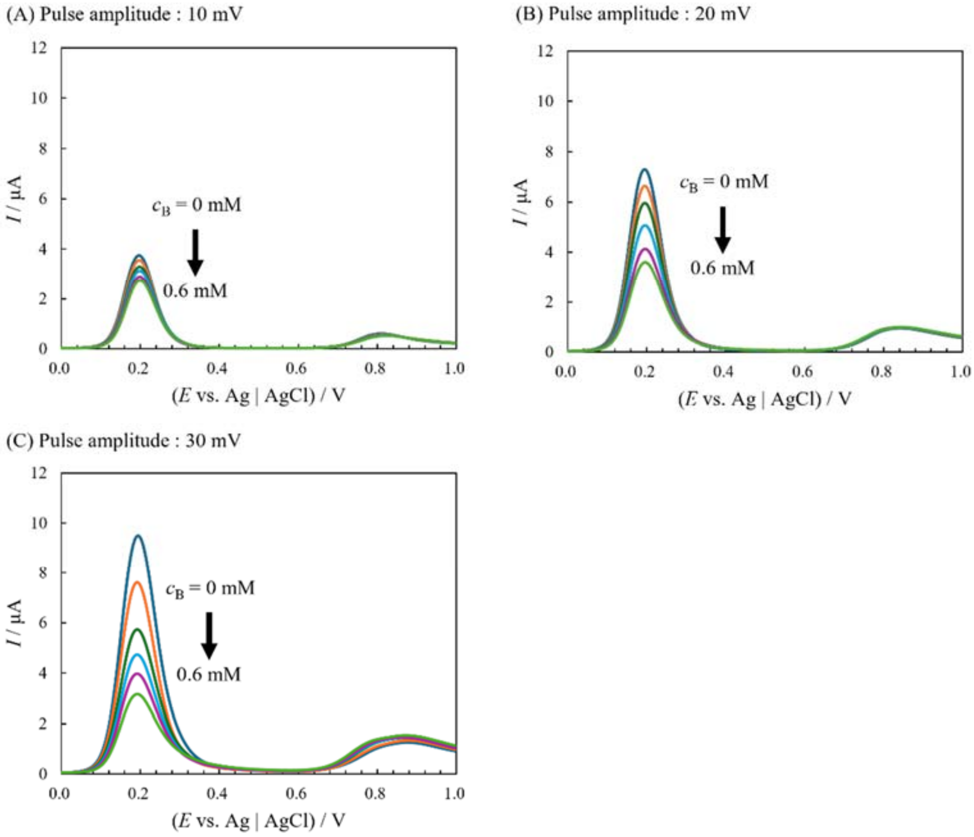

Figure. S35. DPVs of Fc/3,4-DHBA-β-CyD in 90% H_2O/10% CH_3OH (v/v) with various c_B (0 – 0.6 mM) at pH 8.6, r.t. [Fc] = 0.5 mM, [3,4-DHBA-β-CyD] = 1.5 mM, [NaCl] = 0.1 M, [phosphate] = 50 mM. Pulse amplitude = (A) 10, (B) 20, (C) 30 mV, pulse step = 5 mV, a modulation time (pulse width) = 60 ms, interval time (pulse period) of 200 ms, and quiet time = 5 s. WE: sGCDE (ϕ=3.0 mm), RE: Ag|AgCl $_{3M\ NaCl\ aq.}$, CE: Pt coil.

Table S3. I_{pa1} values with various amplitude at various c_B.

Amplitude / mV	c_B / mM	I_{pa1} / μA 1st scan	2nd scan	3rd scan	Average of I_{pa1} / μA	σ / μA
10	0	3.73840	3.69568	3.64380	3.69263	0.03868
	0.1	3.54309	3.47900	3.42712	3.48307	0.04743
	0.2	3.28369	3.24402	3.20740	3.24504	0.03116
	0.3	3.12195	3.08838	3.04260	3.08431	0.03252
	0.4	2.87781	2.87781	2.83203	2.86255	0.02158
	0.6	2.73529	2.64587	2.60498	2.66205	0.05441
20	0	7.29981	7.00684	6.87256	7.05973	0.17839
	0.1	6.63452	6.44531	6.28052	6.45345	0.14464
	0.2	5.96008	5.68848	5.43213	5.69356	0.21557
	0.3	5.07813	4.85840	4.66888	4.86847	0.16722
	0.4	4.13239	3.96301	3.81714	3.97085	0.12882
	0.6	3.59558	3.45062	3.32764	3.45795	0.10951
30	0	9.47693	8.59039	8.13477	8.73403	0.55727
	0.1	7.57935	7.24121	6.83685	7.21914	0.30352
	0.2	5.70557	5.35614	5.05676	5.37282	0.26514
	0.3	4.70154	4.47144	4.24591	4.47296	0.18601
	0.4	3.95294	3.74847	3.52722	3.74288	0.17385
	0.6	3.15308	2.95776	2.80853	2.97312	0.14108

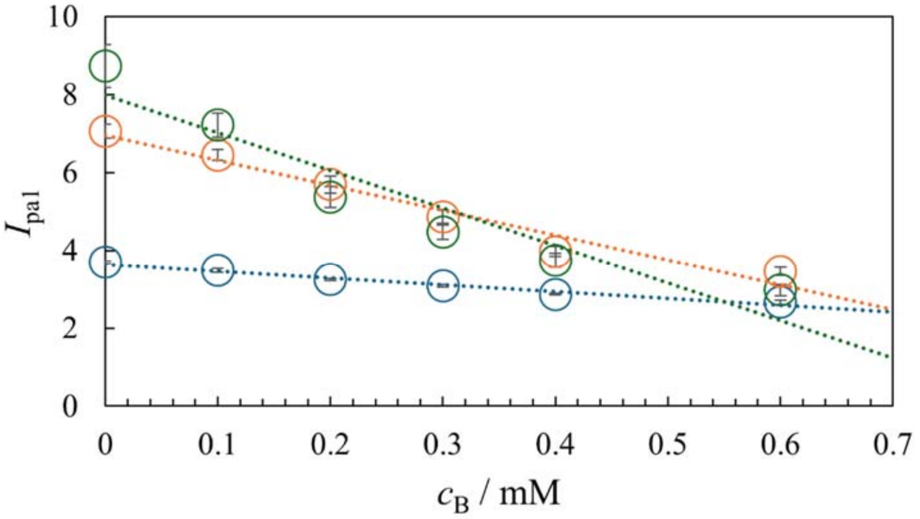

Figure. S36. The relationship between c_B and I_{pa1} (n = 3) at pules amplitude = 10, 20, or 30 mV.

Table S4. The comparison of σ_0, slope and LOD of boron determination using DPV.

Scan rate / mV s^{-1}	σ_0 / μA	Slope / μA M^{-1}	LOD / mg B L^{-1}	R^2
10	0.03868	-1756	0.71	0.9766
20	0.17839	-6400	0.90	0.9641
30	0.55727	-9648	1.87	0.9081

13. SWV response to boron

SWVs of Fc/3,4-DHBA-β-CyD were conducted at various boron concentrations using various parameters. I_{pa1} was decreased with increasing c_B (Figures. S37, S38, and Table S5). LODs were calculated from SWVs measured using various parameters (Table S6). CV at a scan rate of 100 mV s^{-1} was more sensitive than SWV using various parameters.

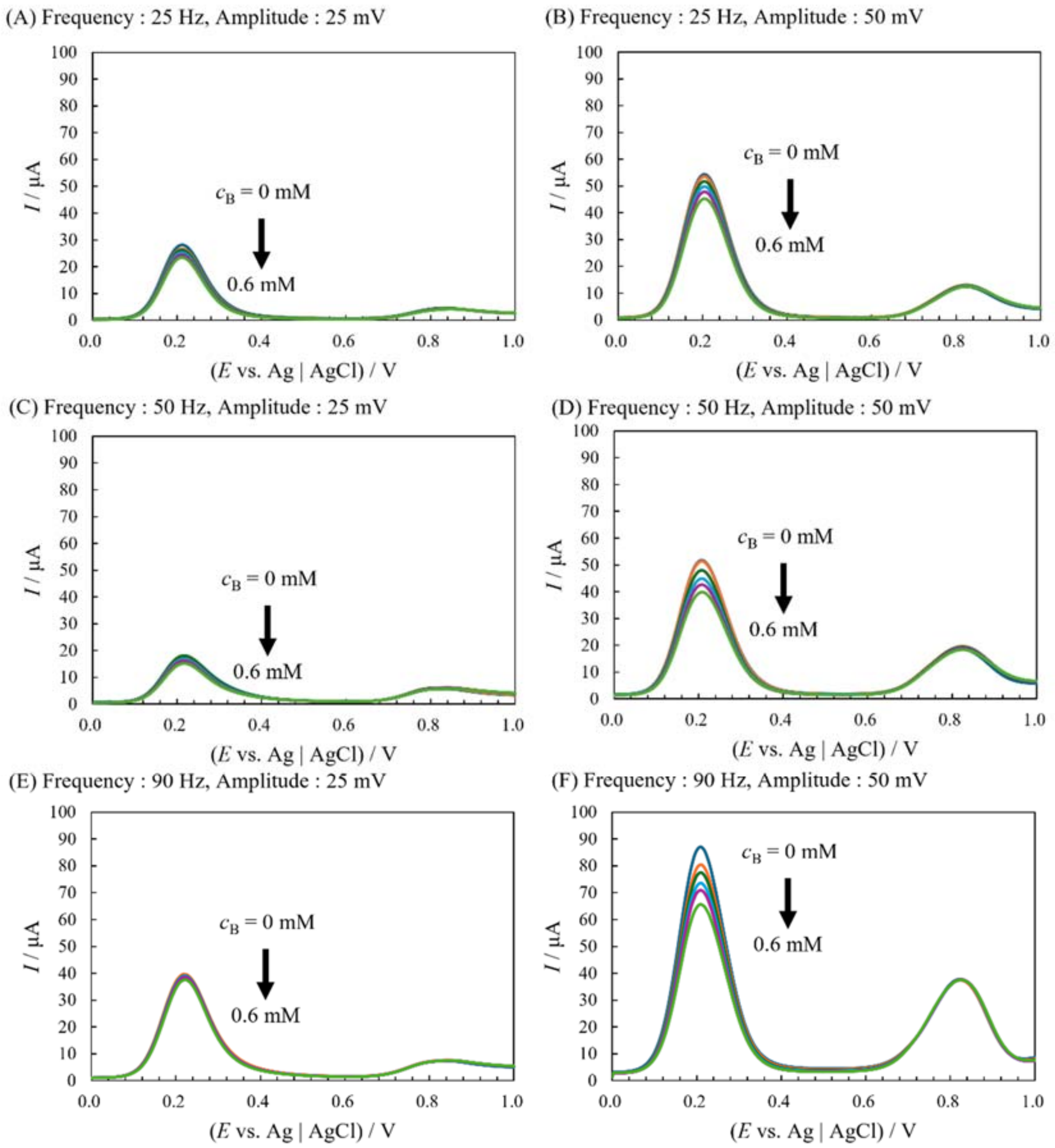

Figure. S37. SWVs of Fc/3,4-DHBA-β-CyD in 90% H_2O/10% CH_3OH (v/v) with various c_B (0 – 0.6 mM) at pH 8.6, r.t. [Fc] = 0.5 mM, [3,4-DHBA-β-CyD] = 1.5 mM, [NaCl] = 0.1 M, [phosphate] = 50 mM. Amplitude = (A), (C), (E) 25 mV or (B), (D), (F) 50 mV, frequency = (A), (B) 25 Hz or (C), (D) 50 Hz or (E), (F) 90 Hz, pulse step = 4 mV, and quiet time = 5 s. WE: sGCDE (ϕ=3.0 mm), RE: Ag|AgCl $_{3M\ NaCl\ aq.}$, CE: Pt coil.

Table S5. I_{pa1} values with various frequency and amplitude at various c_B.

Frequency / Hz	Amplitude / mV	c_B / mM	I_{pa1} / μA 1st scan	2nd scan	3rd scan	Average of I_{pa1} / μA	σ / μA
25	25	0	28.13538	27.67731	27.19879	27.67049	0.38239
		0.1	26.66412	26.56403	26.50452	26.57756	0.06586
		0.2	26.15021	26.01685	25.25879	25.80861	0.39258
		0.3	25.27740	25.13367	24.87213	25.09440	0.16777
		0.4	24.43604	24.18213	23.91052	24.17623	0.21458
		0.6	23.43872	23.14178	23.09662	23.22571	0.15175
	50	0	54.22363	53.69263	53.57056	53.82894	0.28351
		0.1	53.47595	52.57874	52.79236	52.94902	0.38267
		0.2	51.75476	51.16272	50.89417	51.27055	0.35952
		0.3	49.80774	49.36523	48.87390	49.34896	0.38141
		0.4	47.84546	47.48230	47.13135	47.48637	0.29155
		0.6	45.24536	45.11414	44.75708	45.03886	0.20632
50	25	0	17.90680	17.47253	17.31476	17.56470	0.25033
		0.1	18.06458	17.54303	17.64374	17.75045	0.22590
		0.2	18.04199	17.74231	17.51709	17.76713	0.21501
		0.3	16.81061	16.62537	16.34277	16.59292	0.19237
		0.4	16.06903	15.83466	15.60455	15.83608	0.18963
		0.6	15.16022	14.99939	14.89319	15.01760	0.10977
	50	0	51.67542	50.76599	48.63586	50.35909	1.27381
		0.1	51.40991	50.52795	49.76501	50.56763	0.67211
		0.2	47.94922	47.37854	46.74072	47.35616	0.49362
		0.3	44.96155	44.59839	44.13147	44.56380	0.33976
		0.4	42.55371	42.06848	41.58325	42.06848	0.39619
		0.6	39.76135	39.27307	38.76648	39.26697	0.40618
90	25	0	38.68103	38.69934	38.99841	38.79293	0.14549
		0.1	39.56909	39.79187	39.07471	39.47856	0.29970
		0.2	39.04419	39.35852	39.04419	39.14897	0.14818
		0.3	38.99536	38.59863	38.06152	38.55184	0.38267
		0.4	38.59222	37.91473	35.35187	37.28628	1.39552
		0.6	37.59064	37.69348	35.49225	36.92546	1.01430
	50	0	87.00562	85.22644	83.79211	85.34139	1.31442
		0.1	80.51147	79.03137	78.67432	79.40572	0.79536
		0.2	77.48108	76.94092	76.18103	76.86768	0.53326
		0.3	73.62061	73.28491	72.97363	73.29305	0.26419
		0.4	70.86792	70.59326	69.93408	70.46509	0.39186
		0.6	65.70740	64.75220	63.98926	64.81628	0.70289

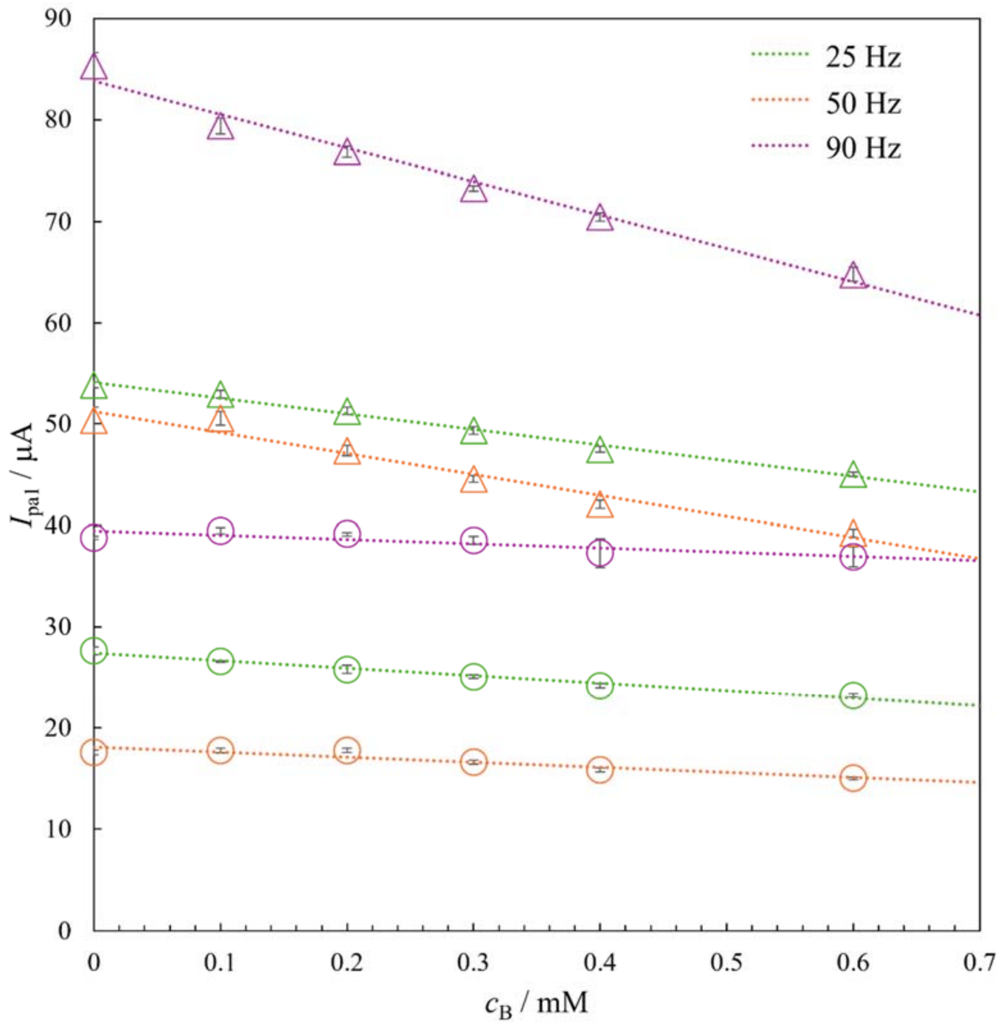

Figure. S38. The relationship between c_B and I_{pa1} of SWV at various parameters ($n = 3$). Amplitude = 25 mV (○) or 50 mV (△).

Table S6. The comparison of σ_0, slope and LOD of boron determination using SWV with various parameters.

Frequency / Hz	Amplitude / mV	σ_0 / μA	Slope / μA M^{-1}	LOD / mg B L^{-1}	R^2
25	25	0.38239	-7402	1.68	0.9816
	50	0.28351	-15462	0.59	0.9904
50	25	0.25033	-4956	1.10	0.8736
	50	1.27381	-20702	2.00	0.9615
90	25	0.14549	-4155	0.23	0.7587
	50	1.31442	-32883	1.30	0.9816

14. Optimization of CV scan rate

CVs were performed at a scan rate of 100, 200, or 400 mV s^{-1} (Figure. S39). The relationship between c_B and I_{pa1} is shown in Figure. S40 and Table S7. LODs were calculated as 0.16, 0.24, and 3.62 mg B L^{-1} at 100, 200, and 400 mV s^{-1}, respectively (Table S8). At 100 mV s^{-1}, LOD had the smallest value, and CV detection of boron was the most sensitive.

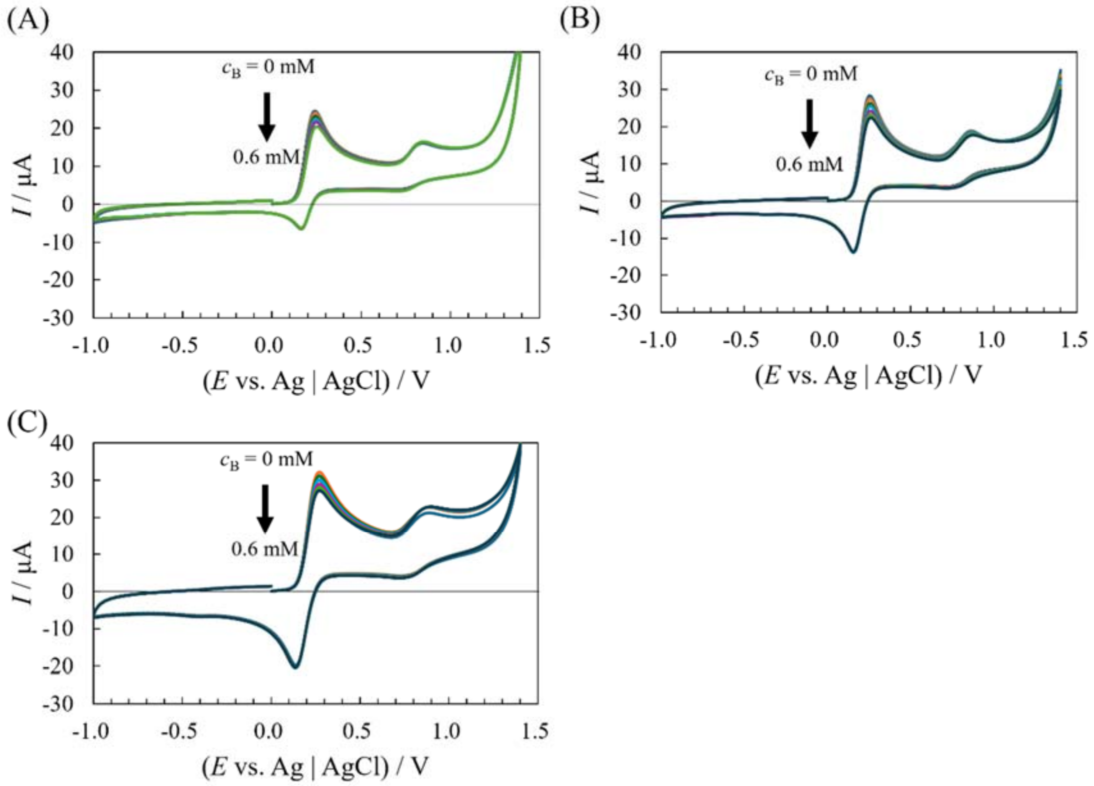

Figure. S39. CVs of Fc/3,4-DHBA-β-CyD with various c_B (0, 0.1, 0.2, 0.3, 0.4, and 0.6 mM) at pH 8.6, r.t. [Fc] = 0.5 mM, [3,4-DHBA-β-CyD] = 1.5 mM, [phosphate] = 50 mM, and [NaCl] = 0.1 M. Scan rate = (A) 100, (B) 200, or (C) 400 mV s^{-1}. WE: sGCDE (ϕ=3.0 mm), RE: Ag|AgCl $_{3M\ NaCl\ aq.}$, CE: Pt coil.

Table S7. I_{pa1} values with various scan rate at various c_B.

Scan rate / mV s^{-1}	c_B / mM	I_{pa1} / μA			Average of I_{pa1} / μA	σ / μA
		1st cycle	2nd cycle	3rd cycle		
100	0	24.52698	24.57581	24.48730	24.53003	0.03620
	0.1	24.02649	23.84949	23.74573	23.87390	0.11591
	0.2	23.21777	23.08044	22.91565	23.07129	0.12351
	0.3	22.36938	22.23816	22.12219	22.24325	0.10098
	0.4	21.60339	21.45386	21.35010	21.46912	0.10397
	0.6	20.41626	20.26978	20.12329	20.26978	0.11960
200	0	28.28674	28.26233	28.11890	28.22266	0.07404
	0.1	27.44141	27.26746	26.98669	27.23185	0.18734
	0.2	26.23596	26.19019	26.22681	26.21765	0.01978
	0.3	25.35706	25.25330	25.20447	25.27161	0.06363
	0.4	24.12109	24.23096	24.05396	24.13534	0.07296
	0.5	23.13538	23.28491	22.89124	23.10384	0.16226
	0.6	22.21069	22.30835	22.30225	22.27376	0.04467
400	0	30.09338	30.75867	32.73621	31.19609	1.12239
	0.1	32.07703	32.01599	31.18286	31.75863	0.40789
	0.2	31.10962	31.02417	31.14624	31.09334	0.05115
	0.3	30.04150	29.44336	29.86755	29.78414	0.25121
	0.4	28.90015	28.59497	29.03137	28.84216	0.18282
	0.5	27.95410	28.11279	28.09753	28.05481	0.07148
	0.6	27.14233	27.21558	27.11792	27.15861	0.04150

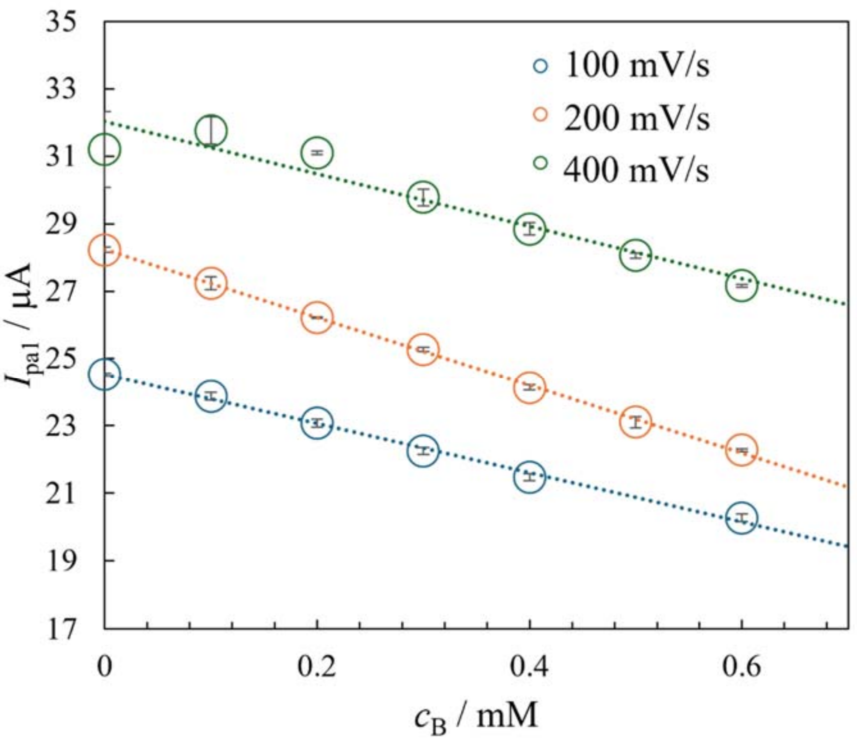

Figure. S40. The relationship between c_B and I_{pa1} of CV at the scan rate of 100, 200, or 400 mVs^{-1} (n = 3).

Table S8. σ_0 and slope of the calibration line, and LOD calculated from them.

Scan rate / mV s^{-1}	σ_0 / μA	Slope / μA M^{-1}	LOD / mg B L^{-1}	R^2
100	0.03620	-7276	0.16	0.9961
200	0.07404	-10066	0.24	0.9991
400	1.12239	-7775	3.62	0.9237
